# Synthesis of Quinoline-Based Rh–Sb Complexes: Inhibition of Halide Transfer to Access a Rh→Sb Z‑Type Interaction

**DOI:** 10.1021/acs.organomet.5c00187

**Published:** 2025-08-01

**Authors:** Xinrui Ou, Fanji Kong, Kevin P. Quirion, Christopher K. Webber, Diane A. Dickie, Daniel H. Ess, T. Brent Gunnoe

**Affiliations:** † Department of Chemistry, 2358 University of Virginia, Charlottesville, Virginia 22904, United States; ‡ Department of Chemistry and Biochemistry, 6756 Brigham Young University, Provo, Utah 84604, United States

## Abstract

We report the synthesis of Rh–Sb complexes using high valent Sb ligands, Q_3_SbCl_2_ (**1**, Q = 8-quinolinyl) and Q_3_SbF_2_ (**2**), from the low valent Rh precursor [(CO)_2_Rh­(μ-Cl)]_2_ to afford the complexes [(κ^4^-Q_3_SbCl)­Rh­(CO)­Cl]­[(CO)_2_RhCl_2_] (**3**) and (κ^4^-Q_3_SbF_2_)­Rh­(CO)Cl (**4**), respectively. The reaction of **1** with [(CO)_2_Rh­(μ-Cl)]_2_ results in the transfer of chloride from Sb to Rh to give the ion pair **3** with a Rh–Sb bond for the cation that, according to computational analysis, has some covalent character. Replacing Sb–Cl with Sb–F bonds (*i.e.*, compound **2**) inhibited halide transfer and allowed formation of **4** with a Rh→Sb interaction that has more Z-type character than the Rh–Sb bond for complex **3**. Molecular orbital and localized orbital bonding analyses are consistent with the proposed Rh→Sb interaction of **4** being more Z-type in character.

## Graphical abstract

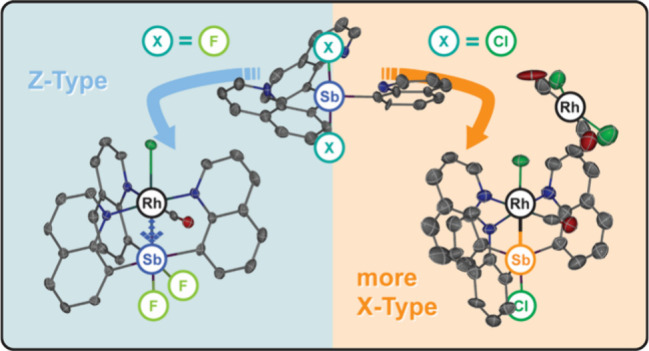

The use of Lewis acidic main group elements as σ-acceptors, or Z-type ligands, has been of recent interest.^1–4^ These efforts have resulted in a broad range of main group elements that have been incorporated as Lewis acidic sites in ligands for transition metal complexes.^2,5^ Over the past two decades, studies have demonstrated the tunable nature of M→Z interactions and impact on catalytic processes using ambiphilic ligands.^4,6,7^ The heavy atom Sb has drawn attention because of its strong fluoride ion affinity and flexible σ-donating/accepting abilities.^8,9^ In addition, Sb has unique coordination and redox noninnocent properties that provide access to different metal–Sb bonding interactions. The Gabbaï group showed that chlorine atoms transfer from Pt to Sb to give Pt complexes with Pt–Sb X-type interactions (Chart 1).^10–12^ Upon substitution of chloride with fluoride, in the presence of cyclohexyl isocyanide (CyNC) or acetonitrile (MeCN), a switch from an X-type interaction to a Z-type interaction between Pt–Sb was proposed.^13^

**Chart 1:**
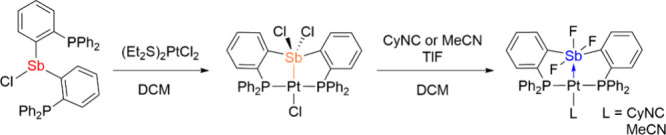
Selected Example of Chlorine Atom Transfer and a Switch of X-Type Interaction to Z-Type Interaction

Our recent work involved the synthesis of a series of Pt–Sb complexes.^14^ We showed that, in some cases, chlorine transfers from Sb to Pt, which impedes access to complexes with Pt→Sb interactions and leads to the formation of product mixtures that complicated purification.^14^ Herein, we report the synthesis of quinoline-based Rh–Sb complexes using high valent Sb^V^ ligands with Sb–F or Sb–Cl bonds, Q_3_SbF_2_ and Q_3_SbCl_2_ (Q = 8-quinolinyl), and their reactions with the low valent Rh^I^ precursor [(CO)_2_Rh­(μ-Cl)]_2_ to afford Rh–Sb complexes featuring X- and Z-type interactions. We show that, by replacing Sb–Cl bonds with Sb–F bonds, halide transfer can be inhibited to give a product with a Rh→Sb interaction.

The reaction of Q_3_SbCl_2_ (**1**, for detailed synthesis and single crystal structure, see , Sections 2 and 4) with [(CO)_2_Rh­(μ-Cl)]_2_ in dichloromethane (DCM) afforded the Rh–Sb complex [(κ^4^-Q_3_SbCl)­Rh­(CO)­Cl]­[(CO)_2_RhCl_2_] (**3**, Scheme 1a). The ^1^H NMR spectrum of **3** shows two characteristic downfield resonances at 11.32 and 10.38 ppm that integrate in a 1:2 ratio, which is consistent with the coordination of three quinoline ligands to the Rh center and the presence of a mirror plane. A crystal suitable for single crystal X-ray diffraction was obtained from vapor diffusion of pentane into a solution of **3** in 1,2-dichloroethane (DCE). Analysis of the crystal structure (Figure 1, left) reveals that 1 equiv of **1** reacts with 1 equiv of the Rh dimer [(CO)_2_Rh­(μ-Cl)]_2_ to give two distinct Rh environments, a cation and an anion that form the ion pair **3**. Regardless of the mechanism, the Sb ligand **1** coordinates to one Rh atom and transfers chloride to a second Rh center to form [(κ^4^-Q_3_SbCl)­Rh­(CO)­Cl]^+^ and [(CO)_2_RhCl_2_]^−^. The IR spectrum of **3** shows v_CO_ at 2091, 2068, and 1982 cm^–1^. The absorptions at 2068 and 1982 cm^–1^ are assigned to the two CO ligands of [(CO)_2_RhCl_2_]^−^. The v_CO_ of this anion has been reported and has ranges 2060–2075 and 1980–1995 cm^–1^.^15–17^ The stretch at 2091 cm^–1^ corresponds to the single terminal CO ligand of [(κ^4^-Q_3_SbCl)­Rh­(CO)­Cl]^+^. The Sb center of **3** adopts a distorted trigonal bipyramidal configuration (τ_5_ = 0.575). In a reported Ni–Sb complex, [{*o*-(Ph_2_P)­C_6_H_4_}_3_SbCl]­NiCl, for which the Sb adopts a similar configuration as **3**, the Sb was described as an X-type ligand with a polarization of electrons toward Ni.^18^ The Rh1–Sb1 bond length of complex **3** is 2.5250(8) Å (Table 1), which is longer than the reported values of 2.4662(13) and 2.4684(7) Å in the related complexes (Q_3_SbCl)­RhCl_2_ and (Q­(Me)_3_SbCl)­RhCl_2_ synthesized by the Gabbaï group.^19^

**Scheme 1:**
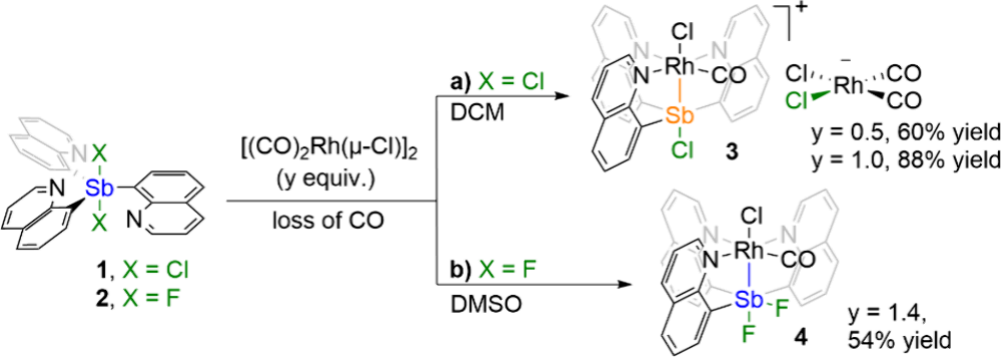
Syntheses of Rh–Sb Complexes [(κ^4^-Q_3_SbCl)­Rh­(CO)­Cl]­[(CO)_2_RhCl_2_] (**3**) and (κ^4^-Q_3_SbF_2_)­Rh­(CO)Cl (**4**)­^a^

a a) for X = Cl, 60% yield when *y* = 0.5 and 88% yield when *y* = 1.0. b) for X = F, the reaction only reaches completion when *y* = 1.4 with 54% yield.

**Figure 1:**
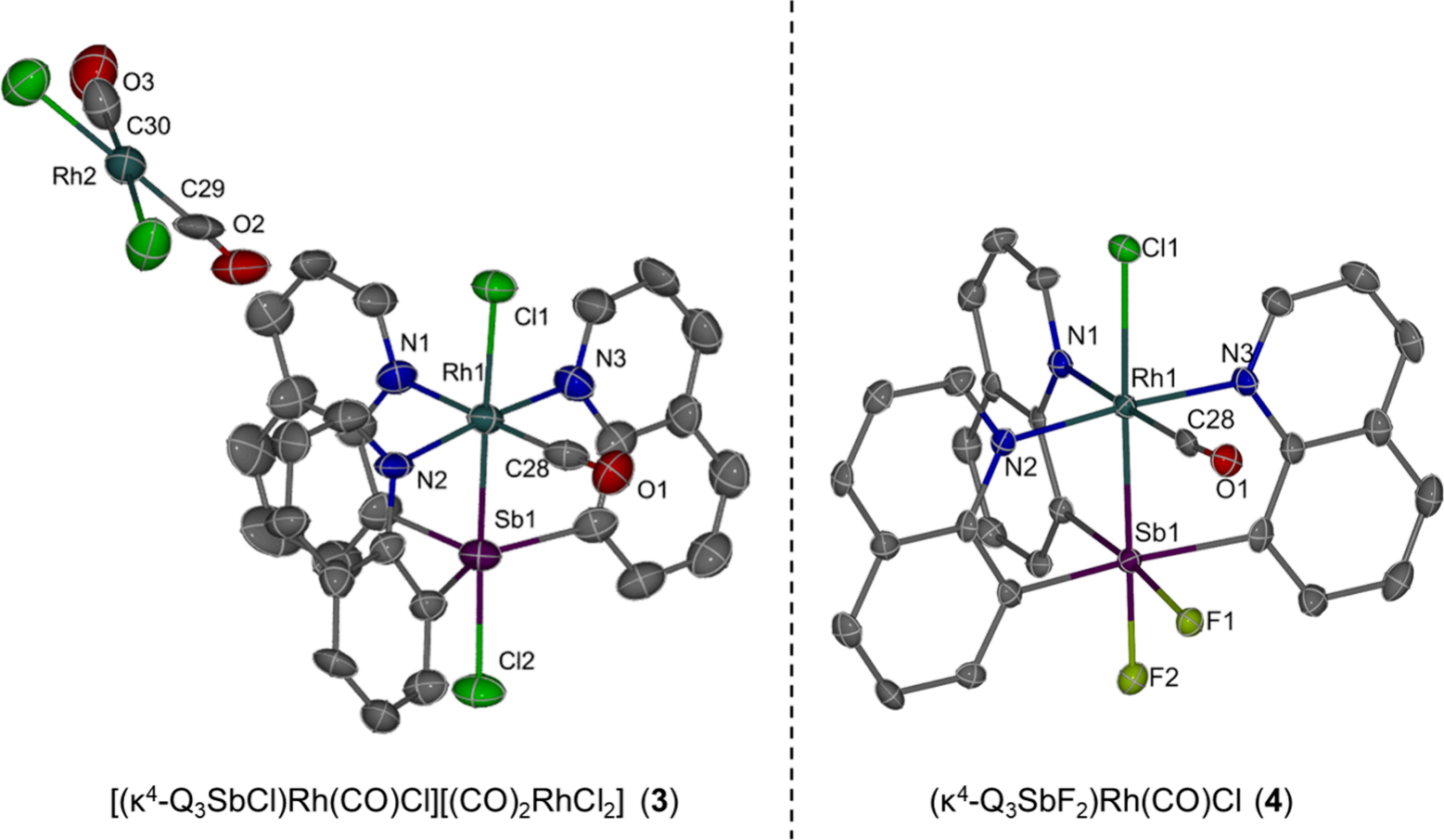
ORTEPs of [(κ^4^-Q_3_SbCl)­Rh­(CO)­Cl]­[(CO)_2_RhCl_2_] (**3**) and (κ^4^-Q_3_SbF_2_)­Rh­(CO)Cl (**4**). Ellipsoids are drawn at the 50% probability level and hydrogen atoms and noncoordinating solvents are omitted for clarity.

**Table 1:** Selected Bond Lengths for Complexes [(κ^4^-Q_3_SbCl)­Rh­(CO)­Cl]­[(CO)_2_RhCl_2_] (**3**) and (κ^4^-Q_3_SbF_2_)­Rh­(CO)Cl (**4**)

**Entry**	**Structural parameter**	**Compound 3** ^a^	**Compound 4**
1	Rh1–Sb1	2.5250(8)	2.5385(5)
2	Rh1–Cl1	2.577(2)	2.6495(14)
3	Rh1–C28	1.885(9)	1.873(6)
4	Rh2–Cl	2.366(3)	
		2.380(5), 2.42(3)	
5	Rh2–C	1.812(18), 1.73(3)	
		1.864(12)	
6	Sb1–Cl2	2.464(2)	
7	Sb1–F1		2.032(3)
8	Sb1–F2		1.984(3)
9	C28–O1	1.081(11)	1.127(7)

a The [(CO)_2_RhCl_2_]^−^ anion in **3** has disordered Rh–Cl and Rh–CO atoms, resulting in two separate bond distances in the structures.

To inhibit halide transfer, we turned our focus to Q_3_SbF_2_ (**2**; for detailed synthesis and single crystal structure, see the , Sections 2 and 4). After optimization, the reaction of Q_3_SbF_2_ with [(CO)_2_Rh­(μ-Cl)]_2_ was carried out in DMSO to give (κ^4^-Q_3_SbF_2_)­Rh­(CO)Cl (**4**, Scheme 1b). The ^1^H NMR spectrum of **4** shows two characteristic downfield resonances at 11.15 and 11.05 ppm that integrate in a 2:1 ratio. The ^19^F­{^1^H} NMR spectrum shows two doublets (^2^ *J*_F,F_ = 43 Hz) at −22.8 and −164.7 ppm. The coupling constant is comparable to the reported ^2^ *J*_F,F_ values of 35–40 Hz for [{*o*-(Ph_2_P)­C_6_H_4_}_2_SbF_3_]­Pt­(NCMe) and [{*o*-(Ph_2_P)­C_6_H_4_}_2_SbF_3_]­Pt­(CNCy) (Cy = cyclohexyl).^13^ A single crystal suitable for an X-ray diffraction study was obtained from vapor diffusion of pentane into a solution of **4** in DCE. As expected, the crystal structure of **4** (Figure 1, right) showed that two fluorides remained bonded to the Sb center. In complex **4**, the Rh1–Sb1 bond length is 2.5385(5) Å, which is comparable to that of **3** (*i.e*., 2.5250(8) Å). The Rh1–Cl1 bond length is 2.6495(14) Å and is significantly longer than that of **3** (2.577(2) Å). This Rh1–Cl1 bond is among the longest reported Rh–Cl bonds in the CCDC database.^20^ The elongation of Rh1–Cl1 bond in **4** is consistent with our observation for Pt–Sb complexes featuring Z-type interactions.^14^ Therefore, similar to our Pt–Sb complexes, the chloride trans to Sb in complex **4** might be better considered as a counterion that is held together via electrostatic interaction with the Rh center. We propose that the interaction between Rh and Sb in **4** is a more Z-type interaction than the Rh–Sb interaction of **3**. The C28–O1 bond length in **4** is 1.127(7) Å and is longer than that in **3** (1.081(11) Å) consistent with the Rh1 center of **4** being more electron-rich than **3**. The v_CO_ for **4** is 2075 cm^–1^ versus 2091 cm^–1^ for **3**. These observations suggest that back-bonding from the Rh center to the CO in **3** is weaker than **4**, which indicates the Rh center in **3** likely has a more positive charge than **4**. In an assignment of oxidation states, likely **4** can be considered closer to Rh^I^, while **3** is closer to Rh^II^, which is consistent with the relative energies of the observed v_CO_ stretches. These observations further support that the Rh–Sb interaction in complex **3** more closely resembles an X-type interaction compared to complex **4**.

To evaluate the bonding interactions between Rh and Sb in complexes **3** and **4**, we used density functional theory (DFT) calculations to generate molecular orbitals (MOs) and localized bonding orbitals. Orbitals were generated from the X-ray structures using the M06-L/def2-TZVP method in Gaussian 16. The quantum theory of atoms in molecules (QTAIM) was used to analyze the bond critical points (BCPs) between Rh and Sb bonds with the Multiwfn^21,22^ software package (see the for additional computational details).

The bonding Rh–Sb MOs (HOMO orbitals) of **3** and **4** are displayed in Figure 2. These MOs are σ-allylic type bonding with contributions from Sb, Rh, and Cl. In **3** the normalized ratio of Rh:Sb contribution is 2:1 and in **4** is 3:1. These ratios are consistent with the orbital bonding being viewed as arising from a dative type of interaction for which there is substantial donation from an electron pair centered on Rh to the empty orbital on Sb. This interaction is significant enough that for **3** the bonding might best be described as verging toward X-type bonding (*i.e*., more covalent) while for **4** the Rh–Sb bonding has more Z-type bonding (*i.e*., less covalent than **3**). Consistent with the HOMO bonding orbital skewing, natural bond orbital (NBO) localization generates a Rh–Sb bonding orbital that is almost entirely localized on the Rh center with very little contribution from Sb, which suggests that, when disconnected, the electron pair is fully localized on the Rh center. Consistent with more skewed bonding in **4** than **3**, the natural population analysis (NPA) charge polarization in **4** is 2.71 compared to 2.29 in **3**.

**Figure 2:**
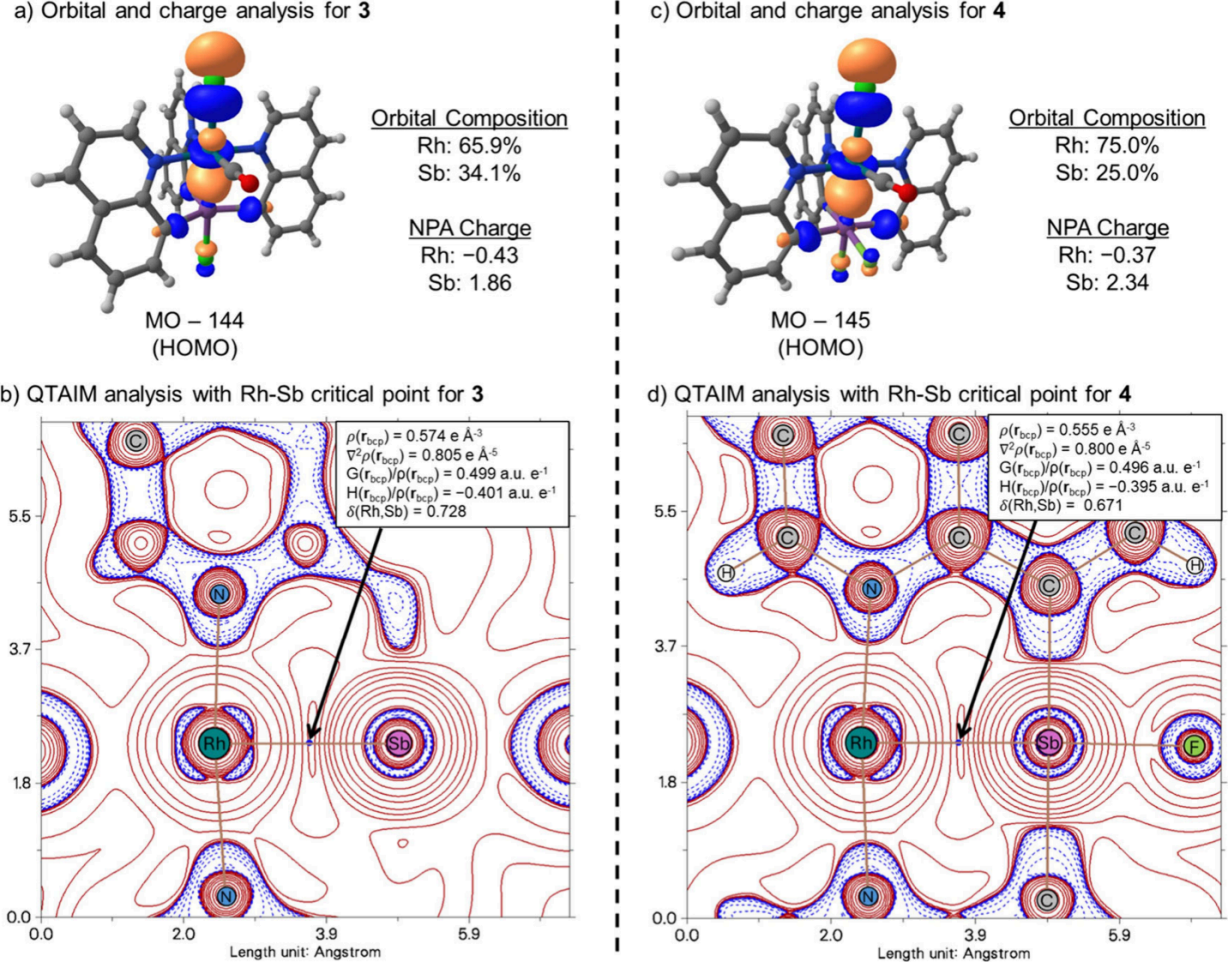
(a) HOMO of **3** including orbital composition and NPA charges for Rh and Sb. (b) QTAIM analysis of the Rh–Sb bond in **3** including the bcp. (c) HOMO of **4** including orbital composition and NPA charges for Rh and Sb. (d) QTAIM analysis of the Rh–Sb bond in **4** including the bcp. For QTAIM, bond critical points (bcp) are shown in blue, and in-plane bonds are shown in brown and plotted as ∇^2^ρ with positive values in red (e^–^ depletion) and negative values in blue (e^–^ concentration).

In addition to MOs, we also inspected the QTAIM points. Most of the QTAIM values are similar for complexes **3** and **4**, such as the Laplacian, kinetic energy density, and electron density at the BCPs. However, there is a slight difference in the delocalization index (δ) values. The δ­(Rh,Sb) value is 0.728 for **3** and δ­(Rh,Sb) = 0.671 for **4**. These values, which are in accord with the MOs built from both Rh and Sb contributions, indicate that, while the Rh–Sb bonding is built from a dative interaction, **3** perhaps has more X-type character than **4**.

In conclusion, we have synthesized quinoline-based Rh–Sb complexes featuring X- and Z-type interactions. The reaction of Q_3_SbCl_2_ with [(CO)_2_Rh­(μ-Cl)]_2_ afforded a Rh–Sb complex (complex **3**) in which the Sb more closely resembles an X-type ligand. This formation of such a complex is due to the transfer of chloride from Sb to Rh. The use of Q_3_SbF_2_ inhibits halide transfer and affords a Rh–Sb complex (complex **4**) featuring a more polarized bond that moves toward a Z-type interaction.

## Supplementary Material





## Notes

Additional experimental procedures and details, NMR spectra, crystal structure data (PDF)
DFT calculations (XYZ)
