# Supplementary material for: Synthesis of Quinoline-Based Rh–Sb Complexes: Inhibition of Halide Transfer to Access a Rh→Sb Z‑Type Interaction
Source: Organometallics. 2025 Aug 1;44(15):1639–43. doi: 10.1021/acs.organomet.5c00187 (PMC12344757; doi:10.1021/acs.organomet.5c00187)
Supplement: Supplementary file 1 [file om5c00187_si_001.pdf]

Supporting Information
for

Synthesis of Quinoline-based Rh–Sb Complexes: Inhibition of Halide Transfer to Access a Rh→Sb Z-type Interaction

Xinrui Ou,[#,a] Fanji Kong,[#,a] Kevin P. Quirion,[b] Christopher K. Webber,[a] Diane A. Dickie,[a] Daniel H. Ess,[b] and T. Brent Gunnoe*,[a]

[a] Department of Chemistry, University of Virginia; Charlottesville, Virginia 22904, United States.

[b] Department of Chemistry and Biochemistry, Brigham Young University, Provo, Utah 84604, United States.

*Corresponding authors. Email: tbg7h@virginia.edu

[#]These authors contributed equally to this work.

Table of Contents

page

1. General Information S3
2. Synthesis and Characterization S4
3. Observed Peak Shifting in ^{1}H NMR of Complex **3** S9
4. Loss of CO in Complex **3** S10
5. NMR Spectra S12
6. IR Spectra S21
7. Crystal Structures of Q_3SbCl_2 and Q_3SbF_2 ....S22
8. X-Ray Crystal Structure Data S23
9. Computational Details and Discussion Based on Resonance Structures S27
10. References S30

1. General Information

Unless otherwise noted, reactions were performed using a dinitrogen-filled glovebox and/or using standard Schlenk techniques. CD_2Cl_2 and $CDCl_3$ (Cambridge Isotopes) was freeze/pump/thaw degassed until no bubbles appeared on thawing (minimum 4 cycles) and stored over 3Å sieves under dinitrogen in a glovebox. DMSO-d_6 (Cambridge Isotopes) and methyl tert-butyl ether were degassed by bubbling dinitrogen for at least half an hour and stored under dinitrogen over 4Å sieves in a glovebox. DCE was distilled from CaH_2 and stored under dinitrogen in a glovebox. Pentanes and benzene were collected from solvent purification system (alumina) and used directly. Iodobenzene dichloride was synthesized as previously reported.[1] Iodobenzene difluoride was synthesized as previously reported.[2] Q_3Sb was synthesized according to the literature.[3] $[(CO)_2Rh(\mu\text{-}Cl)]_2$ was purchased from Sigma-Aldrich with a purity of 97% and stored at -30 °C in a glovebox. All other chemicals were purchased from commercial sources and used as received.

NMR spectra were obtained on a Bruker Avance III 800 MHz or 600 MHz spectrometer or a Bruker Neo Nanobay 400 MHz spectrometer. All reported chemical shifts are referenced to residual ^{1}H resonances (^{1}H NMR) or ^{13}C resonances ($^{13}C\{^{1}H\}$ NMR). ^{1}H NMR: methylene chloride-d_2 5.32 ppm. $^{13}C\{^{1}H\}$ NMR: methylene chloride-d_2 53.84 ppm.[4] ^{19}F NMR spectra were referenced to hexafluorobenzene (δ −164.9 ppm) as an external standard. Infrared spectra were obtained using a Shimadzu IRSpirit-X Compact FT-IR spectrometer. Elemental analyses were performed by the University of Virginia Chemistry Department Elemental Analysis Facility using a Perkin-Elmer CHNS-O series II analyzer.

2. Synthesis and Characterization

Dichloro-tri(quinolin-8-yl)-λ⁵-stibane (**Q_3SbCl_2**, **1**). To a solution of Q_3Sb (500 mg, 0.988 mmol) in 75 mL of DCM (the DCM was kept at -30 °C before use) under dinitrogen, $PhICl_2$ (275 mg, 0.988 mmol) was added portion-wise (100 mg + 100 mg + 75 mg) over 30 mins at room temperature. During the reaction, the flask was covered with aluminum foil. The solution was stirred until it formed a homogeneous light-yellow solution, and then the reaction solution was concentrated to approximately 5 mL followed by slow addition of pentanes. After the formation of a pale-yellow solid, the solid was collected by vacuum filtration. The crude product was then washed with pentanes to yield a pale-yellow/off-white solid product (521 mg, 91% yield). *Note:* the synthesis also works with room temperature DCM with $PhICl_2$ added in one-portion as well as not covering the flask using aluminum foil.

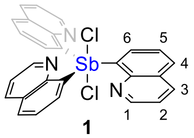

1

^{1}H NMR (600 MHz, CD_2Cl_2) δ 8.73 (d, $^3J_{H,H}$ = 7.2 Hz, 3H, H_1), 8.62 (d, $^3J_{H,H}$ = 3.9 Hz, 3H, H_6), 8.34 (d, $^3J_{H,H}$ = 8.2 Hz, 3H, H_4), 8.13 (d, $^3J_{H,H}$ = 8.0 Hz, 3H, H_3), 7.82 (t, $^3J_{H,H}$ = 7.7 Hz, 3H, H_2), 7.47 (dd, $^3J_{H,H}$ = 8.2, 4.1 Hz, 3H, H_5). $^{13}C\{^1H\}$ NMR (201 MHz, CD_2Cl_2) δ 151.0, 149.8, 146.8, 136.7, 135.7, 130.9, 129.5, 127.2, 122.3. Anal. Calcd for $C_{27}H_{18}N_3SbCl_2 \cdot CH_2Cl_2$: C, 50.80; H, 3.05; N, 6.35. Found: C, 51.04; H, 2.94; N, 6.30. Consistent with the obtained crystal structure that shows one equivalent of DCM per Q_3SbCl_2 (see below, Section 7).

Difluoro-tri(quinolin-8-yl)-λ^5-stibane (**Q_3SbF_2, 2**). To a solution of Q_3Sb (200 mg, 0.395 mmol, 1.0 equiv) in 75 mL of DCM, $PhIF_2$ (120 mg, 0.395 mmol, 1.0 equiv) was added. During the reaction, the flask was covered with aluminum foil. The solution was stirred until it formed a homogeneous light-yellow solution, and then solvent was removed to afford a crude product. The crude product was then washed with diethyl ether and pentanes. The off-white solid was dried under vacuum overnight (145 mg, 67% yield).

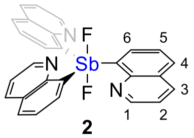

2

^{1}H NMR (600 MHz, $CDCl_3$) δ 8.63 (d, $^3J_{H,H}$ = 4.2 Hz, 3H, H_1), 8.58 (d, $^3J_{H,H}$ = 7.0 Hz, 3H, H_6), 8.25 (d, $^3J_{H,H}$ = 8.2 Hz, 3H, H_4), 8.06 (d, $^3J_{H,H}$ = 8.0 Hz, 3H, H_3), 7.76 (t, $^3J_{H,H}$ = 7.6 Hz, 3H, H_2), 7.38 (dd, $^3J_{H,H}$ = 8.7, 4.2 Hz, 3H, H_5). ^{19}F{^{1}H} NMR (564 MHz, $CDCl_3$) δ −97.5. ^{13}C{^{1}H} NMR (201 MHz, $CDCl_3$) δ 150.2, 148.7, 144.4 (t, $^2J_{F,C}$ = 16 Hz), 136.7, 136.2, 130.6, 129.1, 127.0, 121.7. Anal. Calcd for $C_{27}H_{18}N_3SbF_2$: C, 59.59; H, 3.33; N, 7.72. Found: C, 58.12(17); H, 3.12(7); N, 7.45(2). Standard deviations were calculated from three independent trials.

[(κ^4-Q_3SbCl)Rh(CO)Cl][(CO)$_2$RhCl$_2$] (**3**). <u>Method 1:</u> To a suspension of Q_3SbCl_2 (30 mg, 0.051 mmol) in 3 mL of DCM under dinitrogen, [(CO)$_2$Rh(μ-Cl)]$_2$ (10 mg, 0.0255 mmol) was added. Over time, the suspension changed to an orange homogeneous. After stirring for approximately 1 hour, dry pentanes were added slowly to the solution to precipitate a yellow-orange solid. The solid changed to a slightly darker color after a few minutes in the vial. The solid was collected via vacuum filtration to yield a yellow-tan product (14.4 mg, 60% yield based on Rh dimer). Using a 1:1 mol ratio of Q_3SbCl_2 (30 mg, 0.051 mmol) and [(CO)$_2$Rh(μ-Cl)]$_2$ (20 mg, 0.051 mmol) resulted in a higher yield of complex **3** as a yellow-

orange solid (42 mg, 88% yield). Method 2: To a suspension of Q_3SbCl_2 (60 mg, 0.10 mmol) in 5 mL of DCM under dinitrogen, $[(CO)_2Rh(\mu\text{-}Cl)]_2$ (40 mg, 0.10 mmol) was added. After approximately 15 to 20 mins, the suspension changed to an orange homogeneous solution. Then, dry pentanes were added to the solution to precipitate a yellow-orange solid. The solid was collected via quick vacuum filtration to yield a yellow-tan product (65 mg, 68% isolated yield). Single-crystals for X-ray diffraction were obtained by vapor diffusion of pentanes into a 1,2-dichloroethane solution of isolated product.

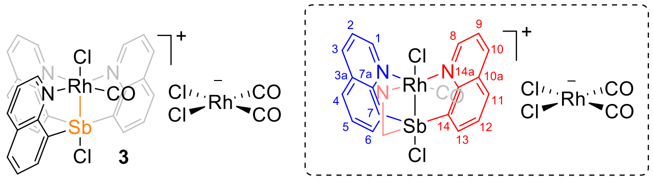
3

^{1}H NMR (400 MHz, $CDCl_3$) δ 11.32 (d, $^3J_{H,H}$ = 5.3 Hz, 1H, H_1), 10.38 (d, $^3J_{H,H}$ = 5.4 Hz, 2H, H_8), 9.48 (d, $^3J_{H,H}$ = 7.5 Hz, 2H, H_{13}), 9.06 (d, $^3J_{H,H}$ = 7.4 Hz, 1H, H_6), 8.78 (d, $^3J_{H,H}$ = 8.2 Hz, 1H, H_3), 8.68 (d, $^3J_{H,H}$ = 8.2 Hz, 2H, H_{10}), 8.24 (d, $^3J_{H,H}$ = 8.0 Hz, 2H, H_{11}), 8.21 (d, $^3J_{H,H}$ = 8.1 Hz, 1H, H_4), 8.03 (dd, $^3J_{H,H}$ = 8.2, 5.4 Hz, 1H, H_2), 7.97 (t, $^3J_{H,H}$ = 7.7 Hz, 2H, H_{12}), 7.76–7.67 (m, 3H, H_5 and H_9). ^{13}C{^{1}H} NMR (201 MHz, $CDCl_3$) δ 181.1 (d, $^1J_{Rh,C}$ = 72 Hz, Rh–*C*O), 179.2 (d, $^1J_{Rh,C}$ = 61 Hz, Rh–*C*O), 159.1 (C_8), 155.8 (C_1, shows as doublet due to the $^1J_{C,H}$ from incomplete ^{1}H decoupling of the proton resonances with chemical shifts > 11 ppm), 149.8 (C_{14a}), 146.8 (C_{7a}), 143.5 (C_3), 142.7 (C_{10}), 142.2 (likely Sb–C_{14}), 140.80 (C_6), 140.75 (C_{13}), 134.3 (C_4), 133.8 (C_{11}), 131.53 (C_{3a}), 131.50 (C_{10a}), 129.2 (C_{12}), 129.0 (C_5), 124.3 (C_2), 123.7 (C_9). The Sb–C_7 carbon resonance is missing due to the broadening effect caused by the quadrupolar Sb nucleus (spin = 5/2 and 7/2). IR(KBr) ν_{CO} = 2091 (Sb–Rh–*C*O), 2064 and 1982 ($[(CO)_2RhCl_2]^-$) cm^{-1}. Method 1: Anal. Calcd for $C_{30}H_{18}N_3O_3SbRh_2Cl_4 \cdot 4CH_2Cl_2$: C, 31.96; H, 2.05; N, 3.29. Found: C, 31.82(19); H, 2.08(3); N, 3.39(10). Standard deviations were calculated from three independent trials.

Method 2: Anal. Calcd for $C_{30}H_{18}N_3O_3SbRh_2Cl_4$: C, 38.42; H, 1.93; N, 4.48. Found: C, 37.35; H, 2.02; N, 4.07. *Note:* the complex is not very stable, storing in the solid-state, in solution, or under vacuum results in loss of CO from the cation and conversion to another complex However, the solid product can be stored within a sealed vial inside the glovebox freezer at −30 °C for a few days without major loss of CO (see Section 4). Method 2: Anal. Calcd for $C_{30}H_{18}N_3O_3SbRh_2Cl_4 \cdot 0.5CH_2Cl_2$: C, 37.37; H, 1.95; N, 4.29. Found: C, 36.42; H, 2.04; N, 3.91.

(κ^4-Q_3SbF_2)Rh(CO)Cl (**4**). Method 1: To a solution of Q_3SbF_2 (50 mg, 0.092 mmol, 1.0 equiv) in DMSO-*d*$_6$ (1.0 mL), $[(CO)_2Rh(\mu\text{-}Cl)]_2$ (50 mg, 0.129 mmol, 1.4 equiv.) was added portion-wise (16 mg + 14 mg + 20 mg) over 70 min at room temperature. Then 5 mL of benzene and 10 mL of methyl tert-butyl ether were added to the solution. A red solid was isolated by filtration. The solid was redissolved in DCM. Cyclohexane was added to precipitate a pink-orange solid (28 mg, 43% yield). Method 2: DMSO (0.5 mL) was added to a solid mixture of Q_3SbF_2 (50 mg, 0.092 mmol, 1.0 equiv) and $[(CO)_2Rh(\mu\text{-}Cl)]_2$ (20 mg, 0.051 mmol, 0.5 equiv). The suspension was stirred for 3 min. Then $[(CO)_2Rh(\mu\text{-}Cl)]_2$ (10 mg, 0.025 mmol, 0.26 equiv) was added to the reaction and stirred for 2 min. The process was repeated for another 2 times ($[(CO)_2Rh(\mu\text{-}Cl)]_2$ in total 50 mg, 1.4 equiv). Anhydrous benzene (3 mL) was added slowly to precipitate the crude product. The crude product was washed with benzene and then redissolved in DCM. Pentane was added to precipitate the product (35 mg, 54% isolated yield). Note: The product decomposes under vacuum overnight, possibly due to the loss of CO.

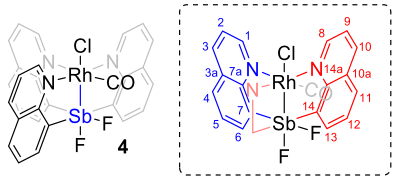

^{1}H NMR (600 MHz, CD_2Cl_2) δ 11.15 (br s, 2H, H_8), 11.05 (br s, 1H, H_1), 8.86 (d, $^{3}J_{H,H}$ = 6.9 Hz, 2H, H_{13}), 8.61 (d, $^{3}J_{H,H}$ = 7.0 Hz, 1H, H_6), 8.31 (d, $^{3}J_{H,H}$ = 8.1 Hz, 2H, H_{10}), 8.17 (d, $^{3}J_{H,H}$ = 8.1 Hz, 1H, H_3), 7.80 (d, $^{3}J_{H,H}$ = 7.8 Hz, 2H, H_{11}), 7.73 (t, $^{3}J_{H,H}$ = 7.5 Hz, 2H, H_{12}), 7.56 (d, $^{3}J_{H,H}$ = 7.5 Hz, 1H, H_4), 7.53 (t, $^{3}J_{H,H}$ = 6.7 Hz, 1H, H_2), 7.48 (t, $^{3}J_{H,H}$ = 6.3 Hz, 2H, H_9), 7.44 (t, $^{3}J_{H,H}$ = 7.5 Hz, 1H, H_5). ^{13}C{^{1}H} NMR (201 MHz, CD_2Cl_2) δ 182.1 (d, $^{1}J_{Rh,C}$ = 63 Hz, Rh–*C*O), 159.8 (C_8), 157.5–157.0 (m, Sb–C_7), 156.0–155.3 (m, Sb–C_{14}), 154.7 (C_1), 147.5 (C_{14a}), 146.3 (C_{7a}), 141.22 (C_{10} or C_3), 141.21 (C_{10} or C_3), 137.9 (C_6), 136.6 (C_{13}), 131.0 (C_{10a}), 130.4 (C_{3a}), 129.4 (C_{11}), 128.8 (C_4), 128.53 (C_{12}), 128.48 (C_5), 122.3 (C_2), 122.2 (C_9). ^{19}F{^{1}H} NMR (377 MHz, CD_2Cl_2) δ −22.8 (d, $^{2}J_{F,F}$ = 43 Hz), −164.7 (d, $^{2}J_{F,F}$ = 43 Hz). IR(KBr) ν_{CO} = 2075 cm^{-1} (Sb–Rh–*C*O). Method 1: Anal. Calcd for: $C_{28}H_{18}ClF_2N_3ORhSb\cdot0.3DMSO\cdot0.17C_6H_{12}$: C, 47.53; H, 2.94; N, 5.62. Found: C, 47.00; H, 3.16; N, 4.84. Method 2: Anal. Calcd for: $C_{28}H_{18}ClF_2N_3ORhSb\cdot CH_2Cl_2$: C, 43.79; H, 2.53; N, 5.28. Found: C, 42.92; H, 2.43; N, 5.13.

3. Observed Peak Shifting in ^1H NMR of Complex 3

As shown in **Figure S1**, the proton resonances of [(κ^4-Q_3SbCl)Rh(CO)Cl][$(CO)_2RhCl_2$] (**3**) were found to be slightly shifted between each NMR acquisition, which is potentially related to: (a) the residual DCM from the synthesis, (b) the concentration of complex **3** in solution, or (c) the formation of small amounts of other complex (*e.g.*, loss CO) during the removal of DCM.

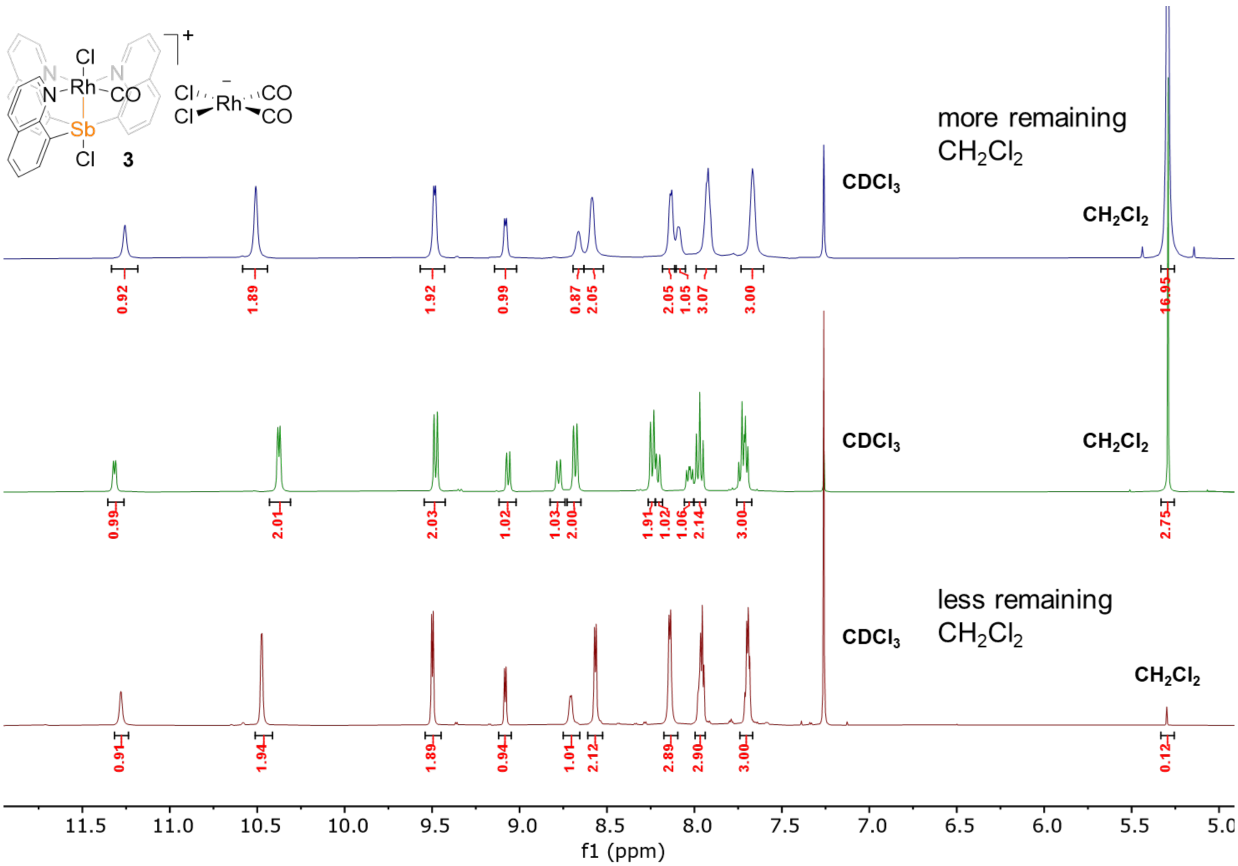

Figure S1. ^1H NMR spectra of [(κ^4-Q_3SbCl)Rh(CO)Cl][$(CO)_2RhCl_2$] (**3**) in $CDCl_3$ with different amounts of remaining CH_2Cl_2 from the synthesis.

4. Loss of CO in Complex 3

Drying complex **3** under vacuum results in the formation of unknown complex (**Figure S2**). The unknown complex can be isolated by reprecipitation from the $CDCl_3$ solution with pentanes or slow evaporation of the $CDCl_3$ solution. The isolated yellow solid product can be redissolved in DCM or chloroform (**Figure S3**), which is distinct from the related insoluble Sb–Rh complex reported by Wade and Gabbaï.[5] The ν_{CO} band at 2091 cm^{-1} of **3** disappeared in the solid-state IR spectrum of the unknown complex (**Figure S4**), which suggests the loss of CO from the cationic moiety. Therefore, we speculated that complex **3** converts to a dimeric or monomeric anionic complex after loss CO, instead of a chloride transfer from the $[(CO)_2RhCl_2]^-$ anion to displacing the CO ligand to from the Wade/Gabbaï complex.

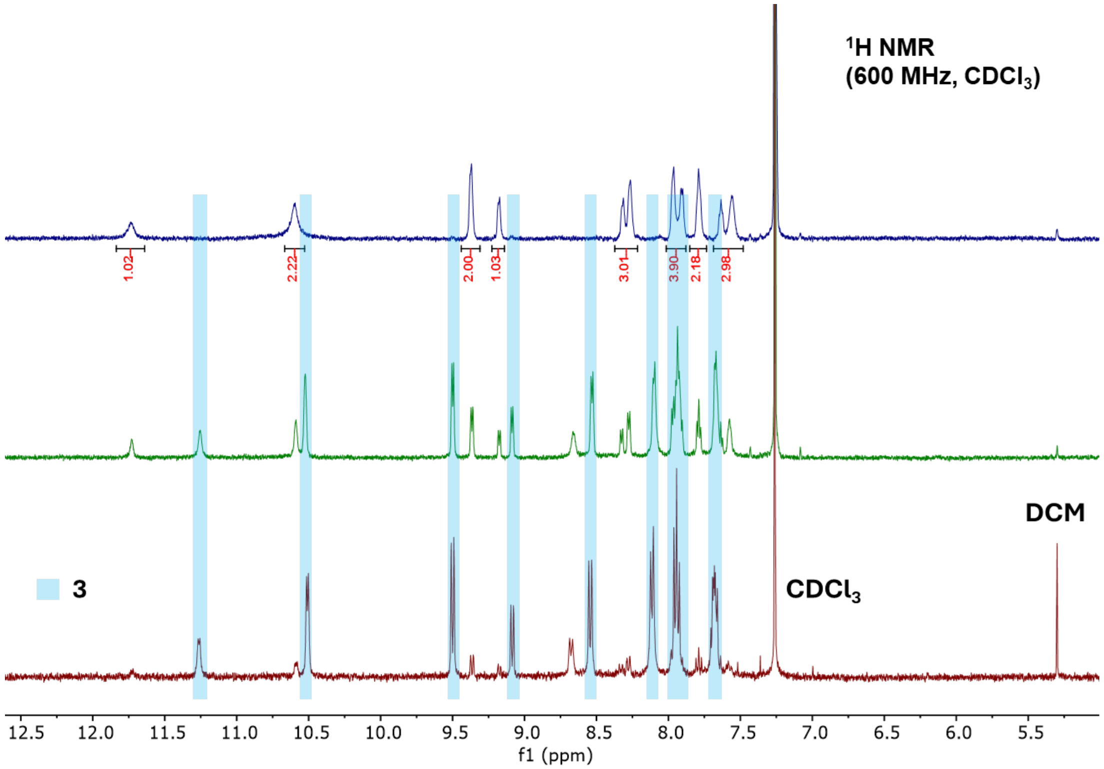

Figure S2. ^{1}H NMR spectra of the conversion of complex 3 to an unidentified complex.

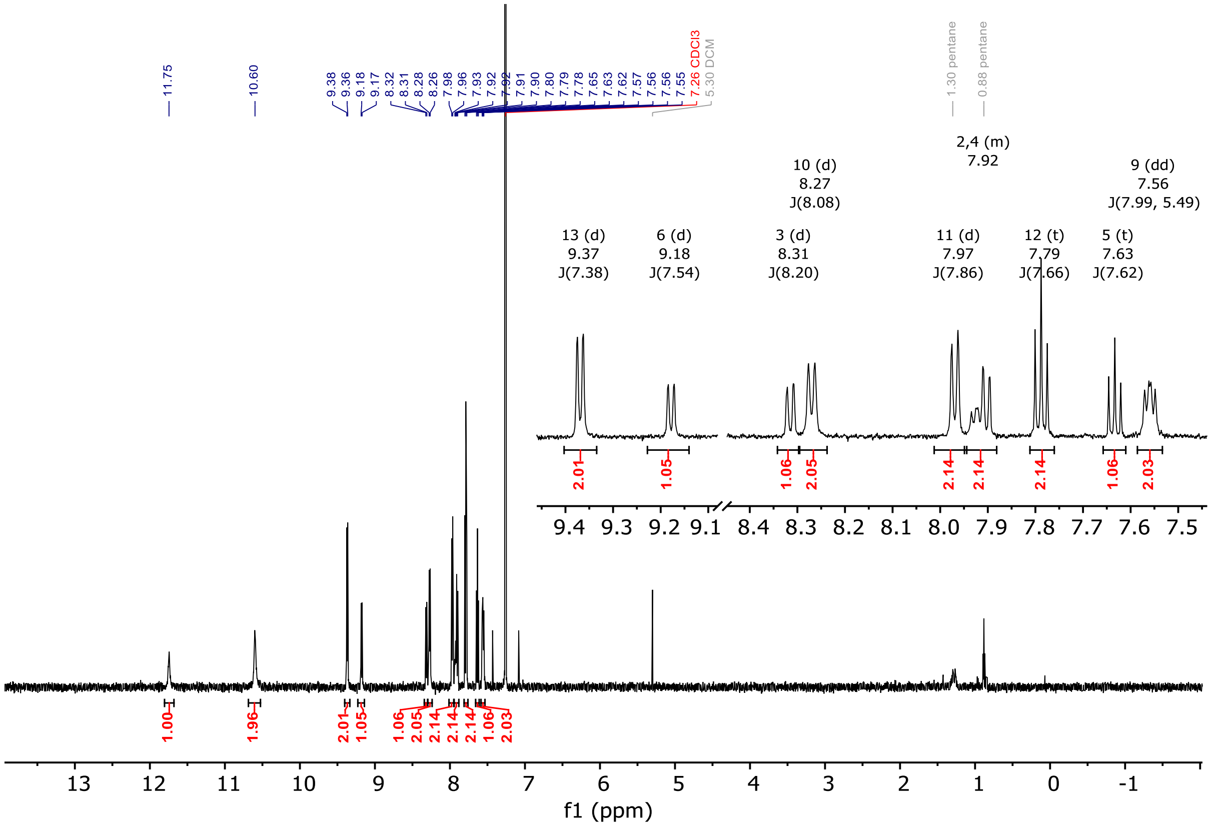

Figure S3. ^{1}H NMR spectrum of the isolated unknown complex.

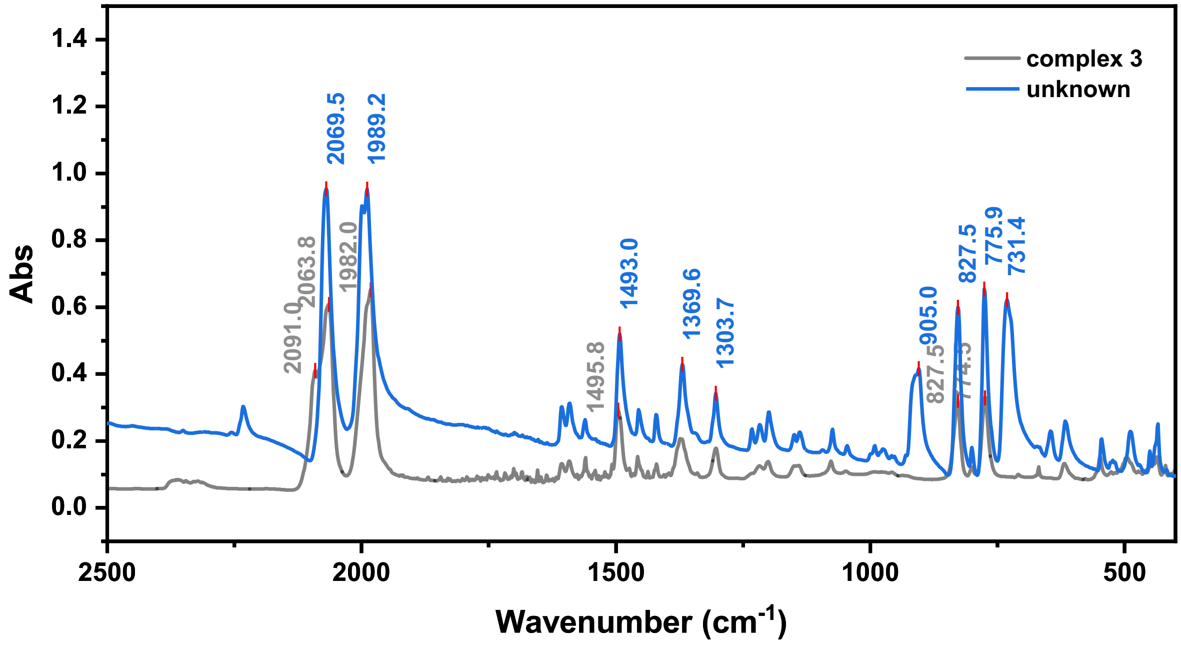

Figure S4. IR Spectra of unknown complex and complex **3**.

5. NMR Spectra

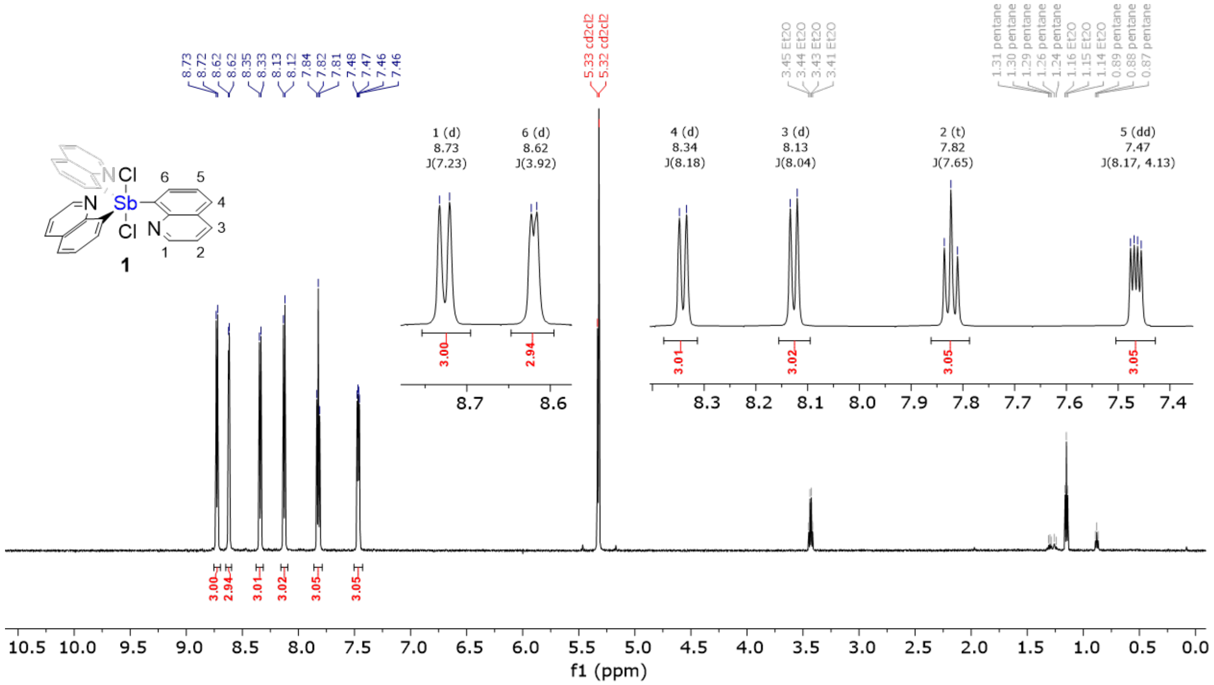

Figure S5. ^{1}H NMR spectrum of Q_3SbCl_2 (**1**) (600 MHz, CD_2Cl_2).

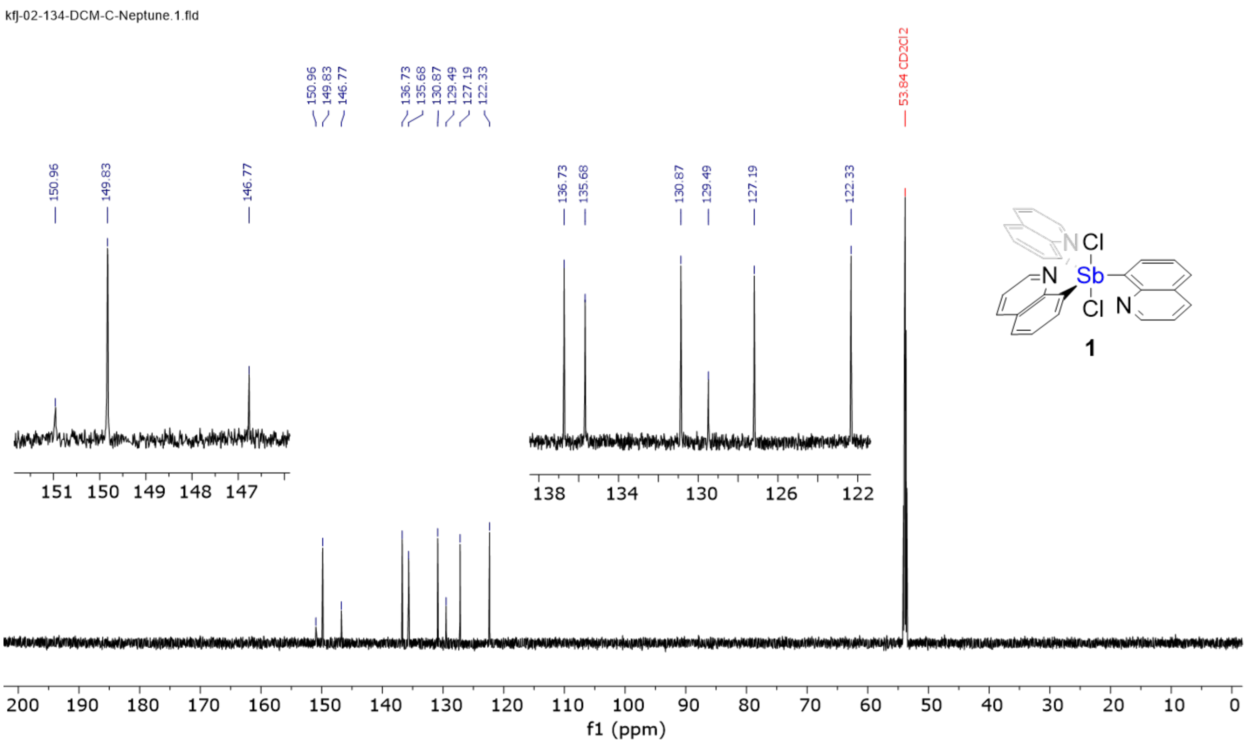

Figure S6. $^{13}C\{^1H\}$ NMR spectrum of Q_3SbCl_2 (**1**) (201 MHz, CD_2Cl_2).

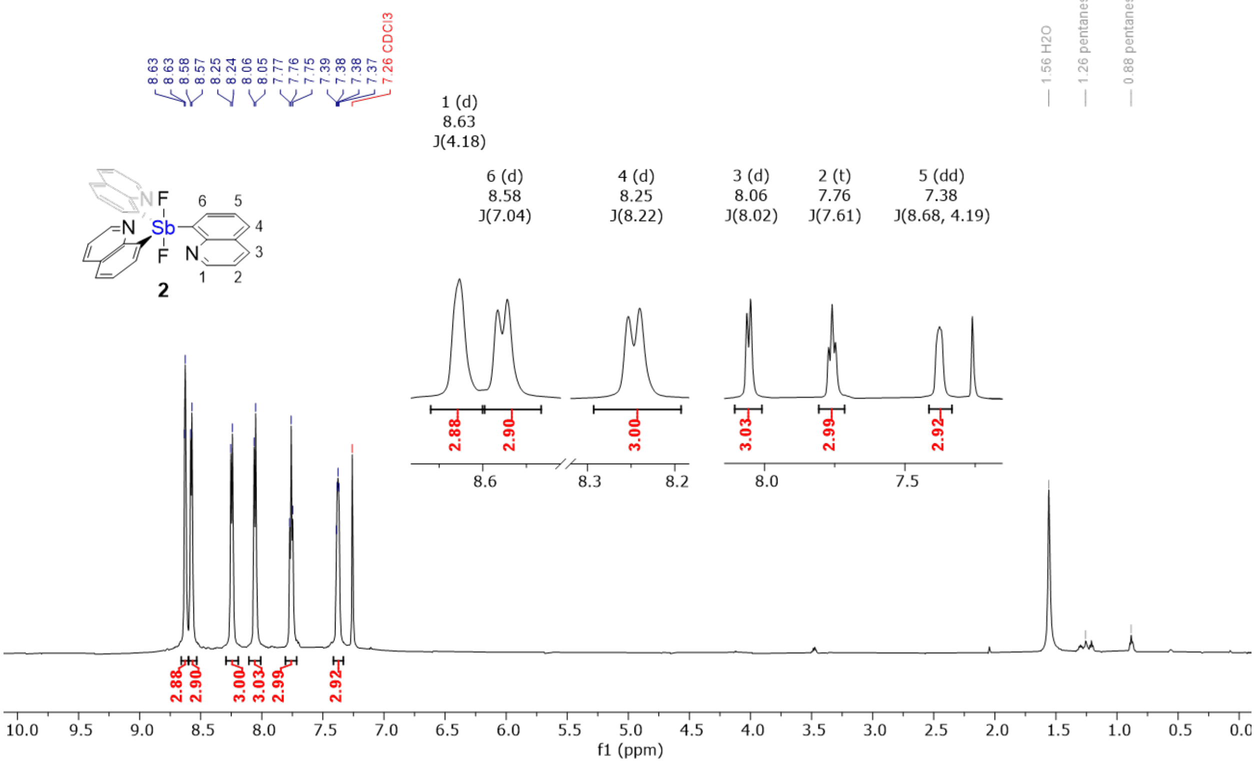

Figure S7. ^{1}H NMR spectrum of Q_3SbF_2 (**2**) (600 MHz, $CDCl_3$).

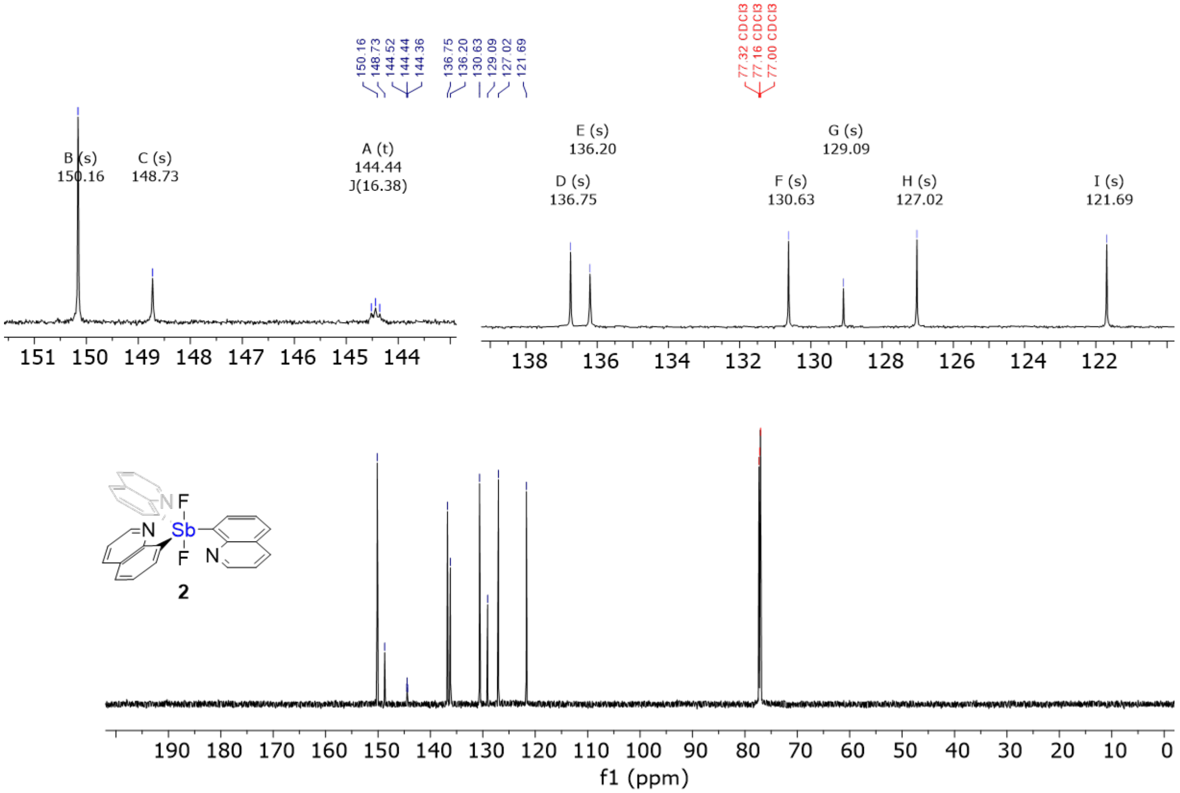

Figure S8. $^{13}C\{^{1}H\}$ NMR spectrum of Q_3SbF_2 (**2**) (201 MHz, $CDCl_3$).

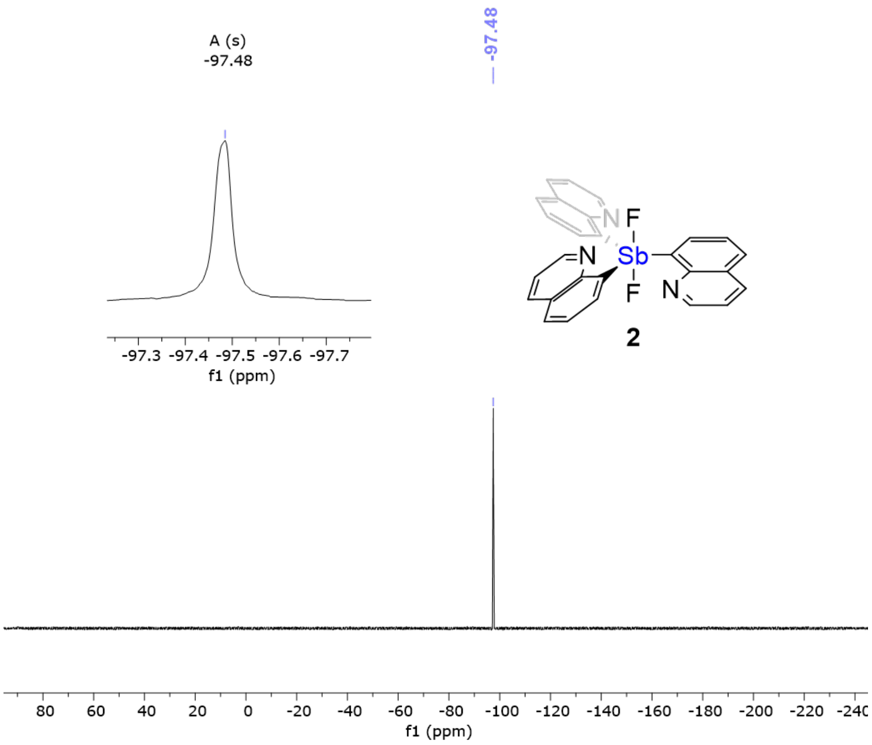

Figure S9. $^{19}F\{^1H\}$ NMR spectrum of Q_3SbF_2 (**2**) (564 MHz, $CDCl_3$).

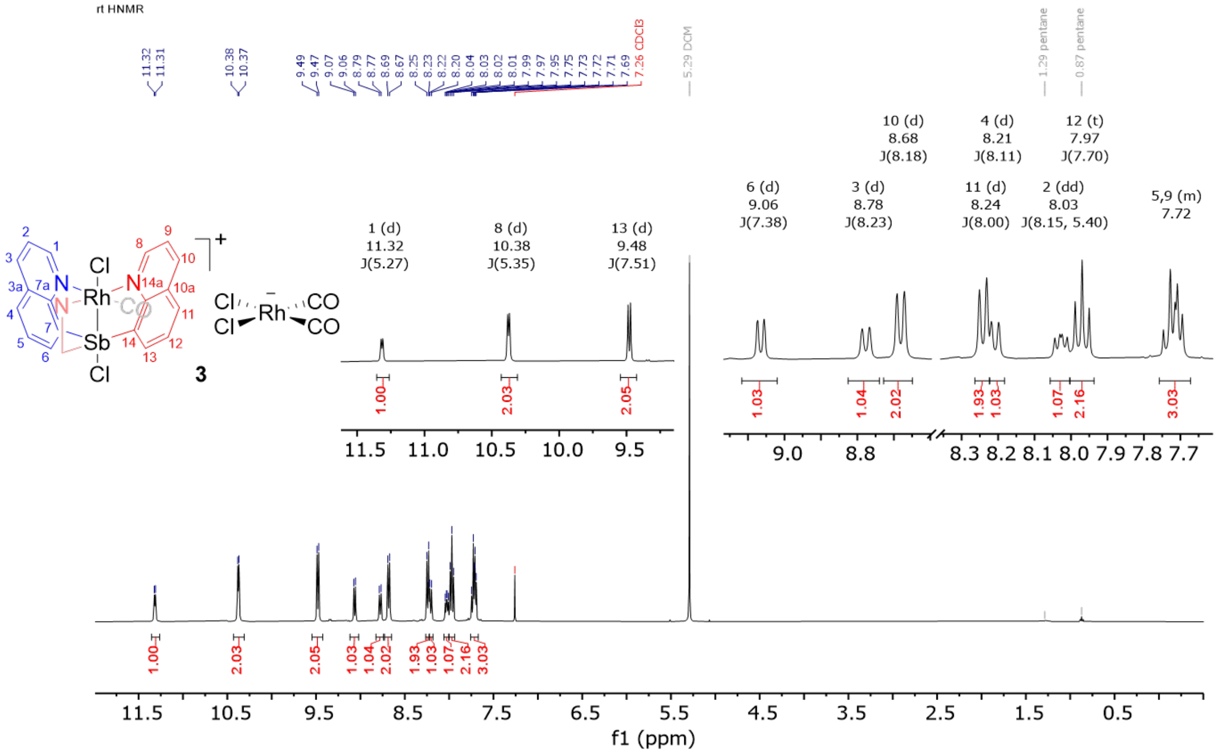

Figure S10. ^1H NMR spectrum of $[(\kappa^4\text{-}Q_3SbCl)Rh(CO)Cl][(CO)_2RhCl_2]$ (**3**) (400 MHz, $CDCl_3$).

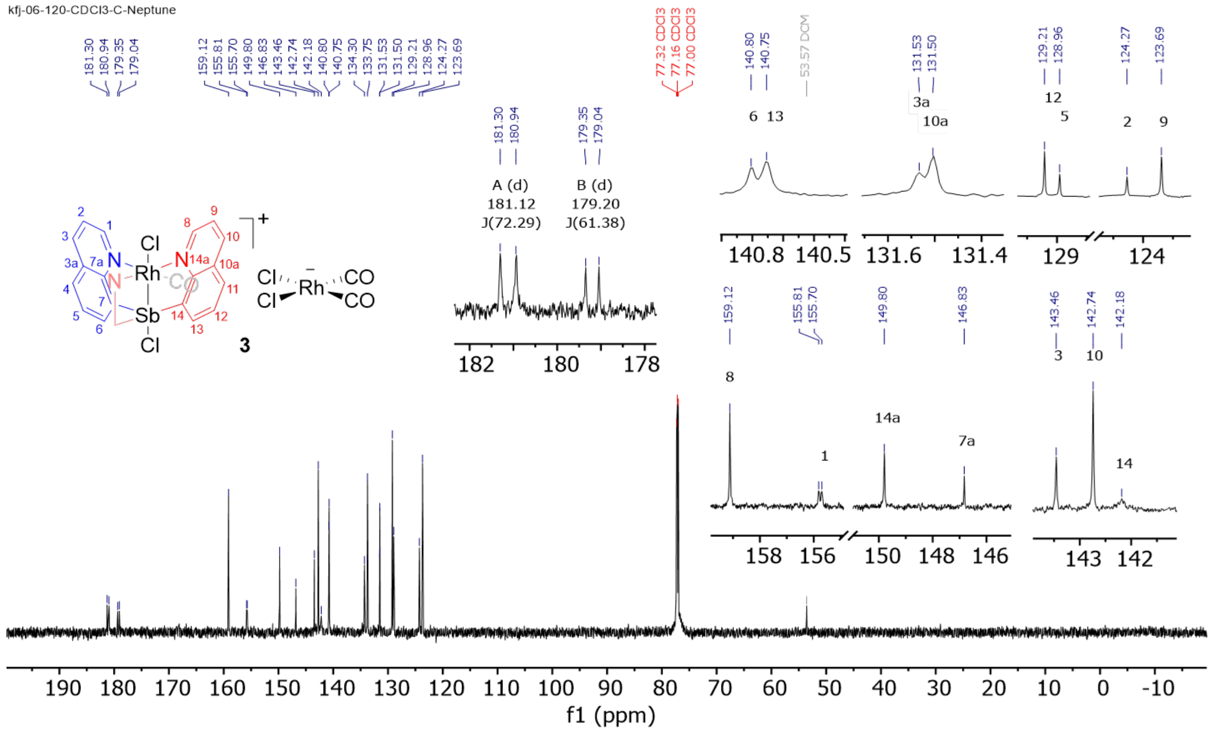

Figure S11. $^{13}C\{^{1}H\}$ NMR spectrum of [(κ^4-Q_3SbCl)Rh(CO)Cl][(CO)$_2RhCl_2$] (**3**) (201 MHz, $CDCl_3$).

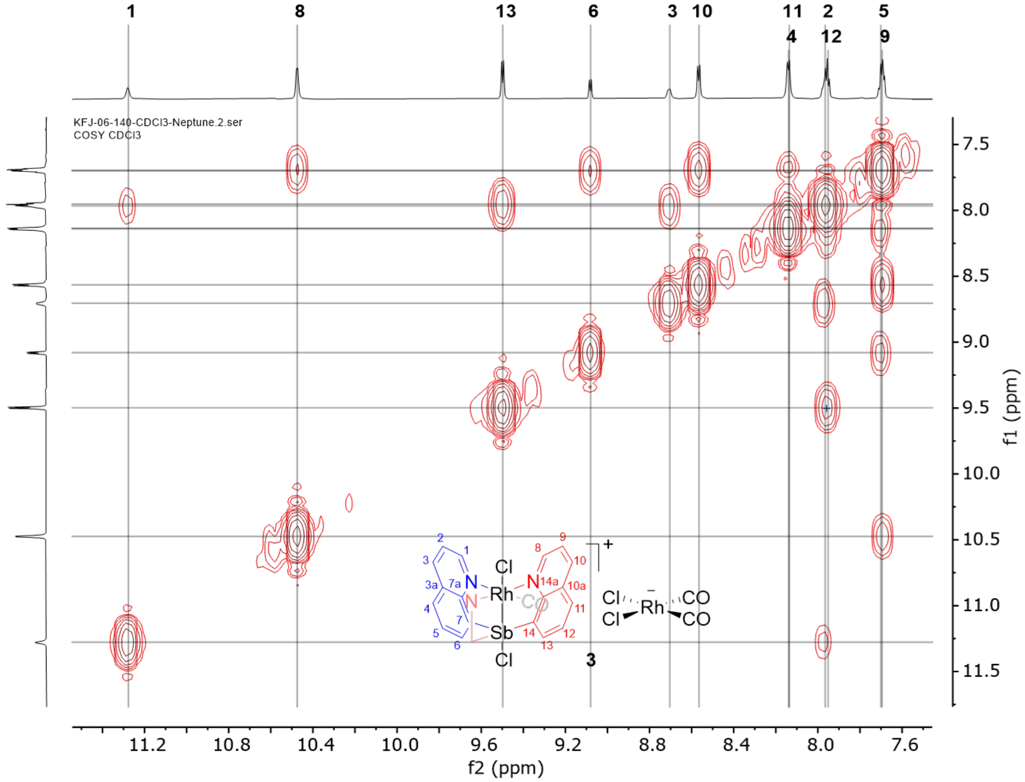

Figure S12. COSY NMR spectrum of [(κ^4-Q_3SbCl)Rh(CO)Cl][(CO)$_2RhCl_2$] (**3**) (800 MHz, $CDCl_3$).

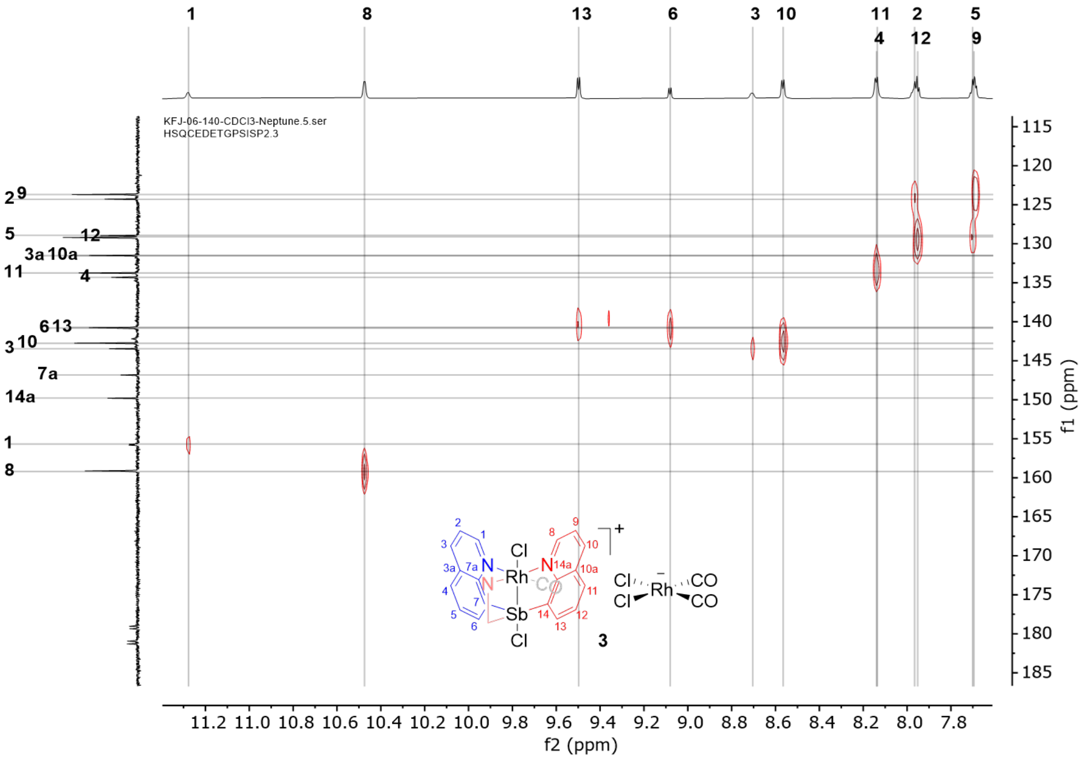

Figure S13. HSQC NMR spectrum of [(κ^4-Q_3SbCl)Rh(CO)Cl][$(CO)_2RhCl_2$] (**3**) (800, 201 MHz, $CDCl_3$).

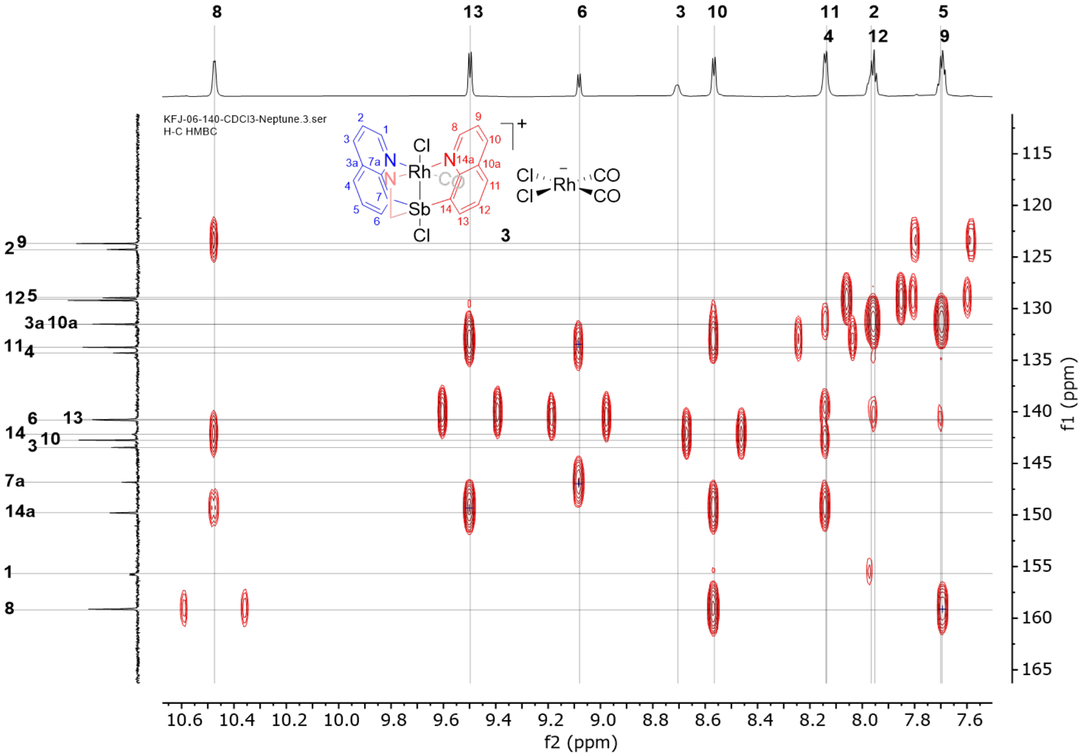

Figure S14. HMBC NMR spectrum of [(κ^4-Q_3SbCl)Rh(CO)Cl][$(CO)_2RhCl_2$] (**3**) (800, 201 MHz, $CDCl_3$).

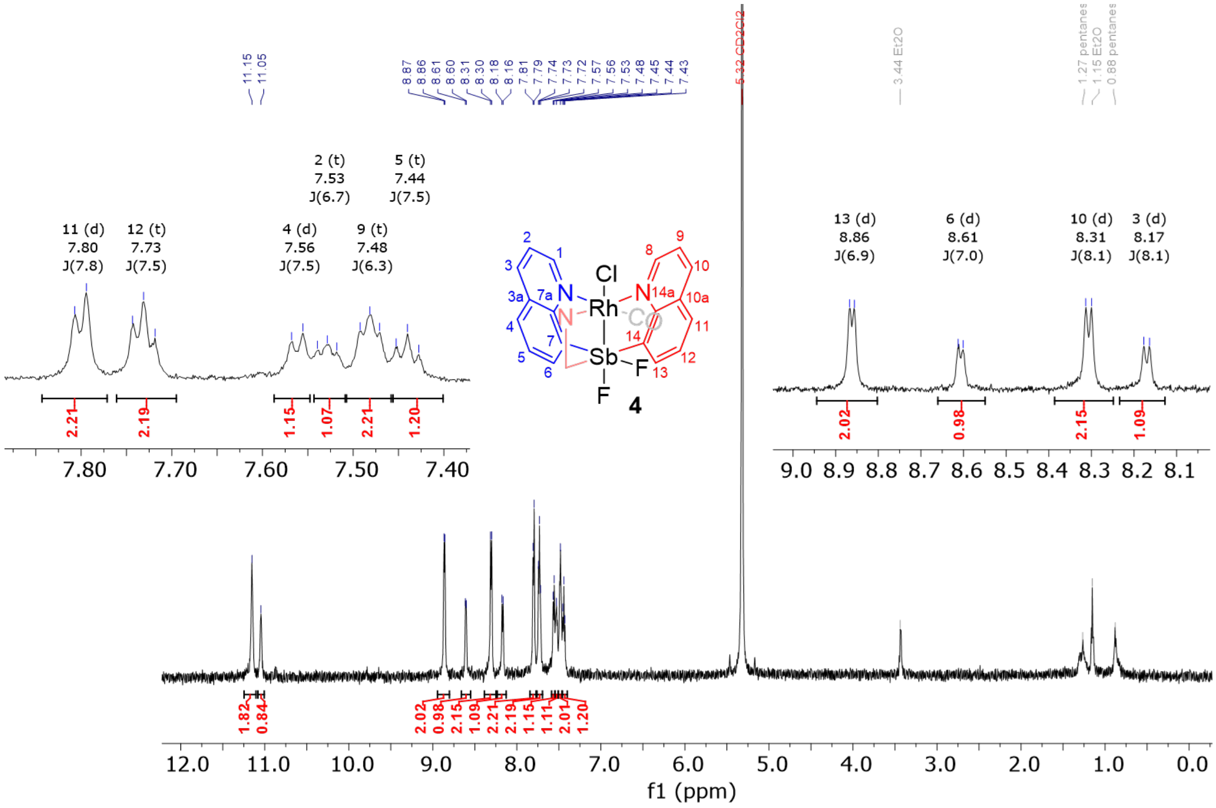

Figure S15. ^{1}H NMR spectrum of (κ^4-Q_3SbF_2)Rh(CO)Cl (**4**) (600 MHz, CD_2Cl_2).

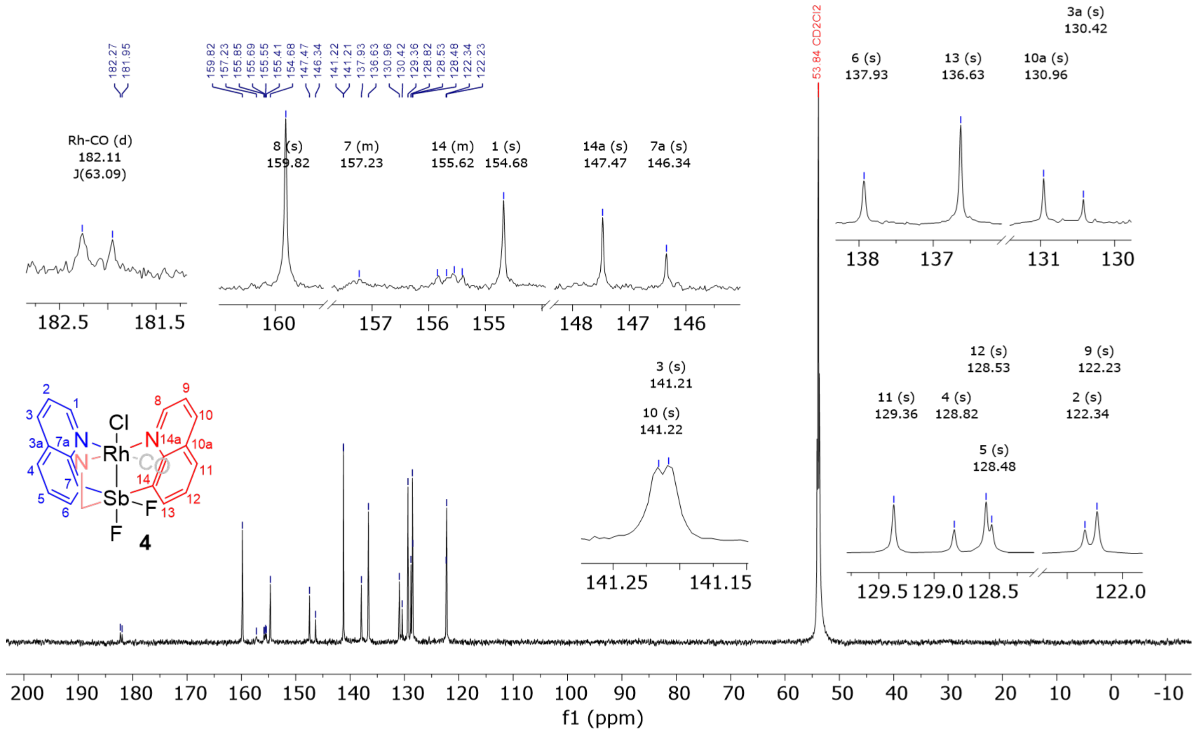

Figure S16. $^{13}C\{^{1}H\}$ NMR spectrum of (κ^4-Q_3SbF_2)Rh(CO)Cl (**4**) (201 MHz, CD_2Cl_2).

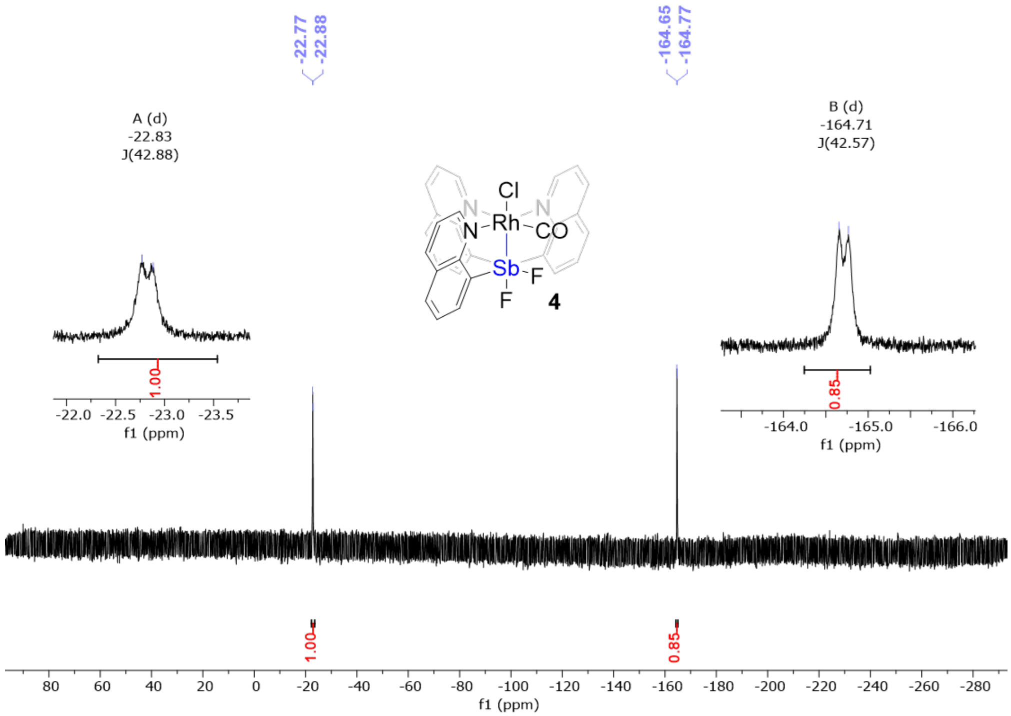

Figure S17. $^{19}F\{^{1}H\}$ NMR spectrum of (κ^4-Q_3SbF_2)Rh(CO)Cl (**4**) (377 MHz, CD_2Cl_2).

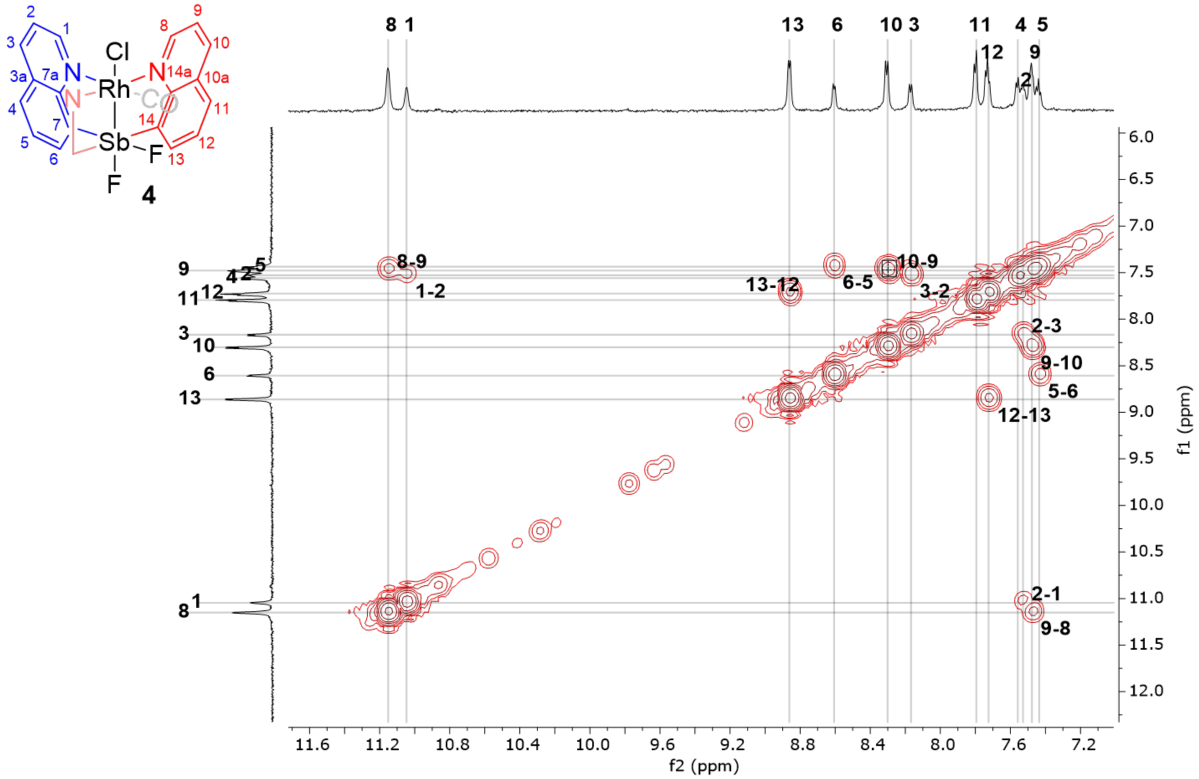

Figure S18. COSY NMR spectrum of (κ^4-Q_3SbF_2)Rh(CO)Cl (**4**) (800 MHz, CD_2Cl_2).

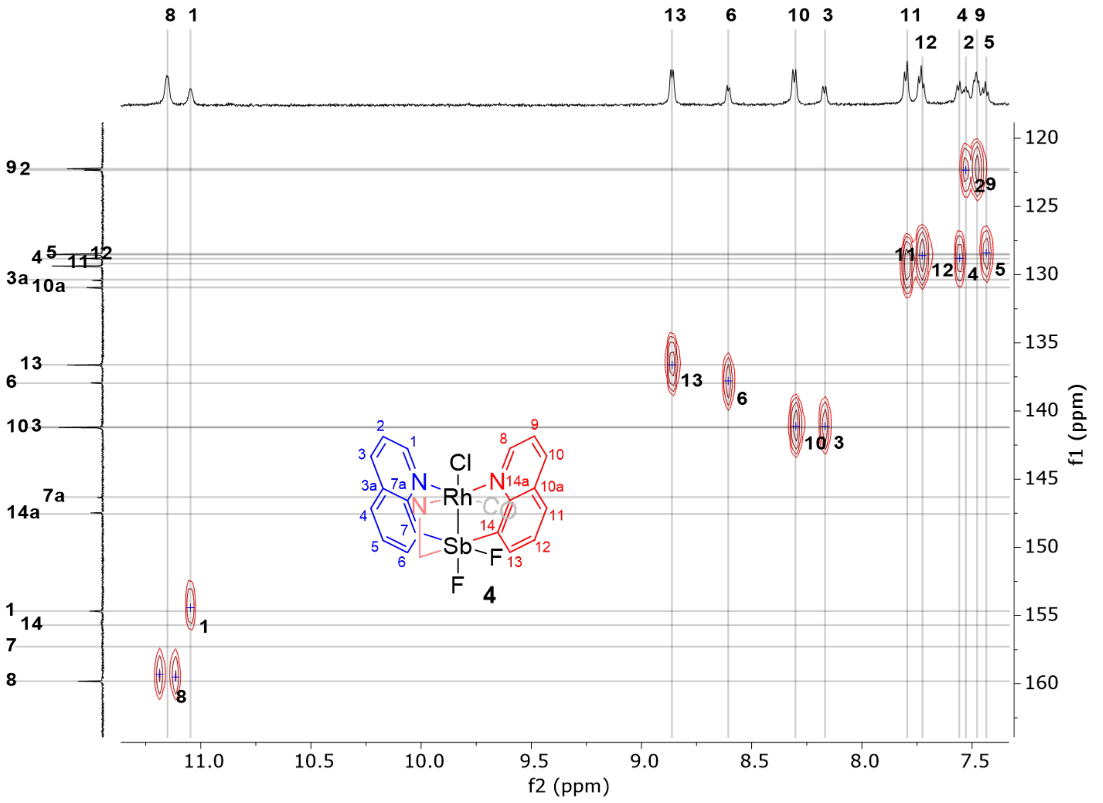

Figure S19. HSQC NMR spectrum of (κ^4-Q_3SbF_2)Rh(CO)Cl (**4**) (800, 201 MHz, CD_2Cl_2).

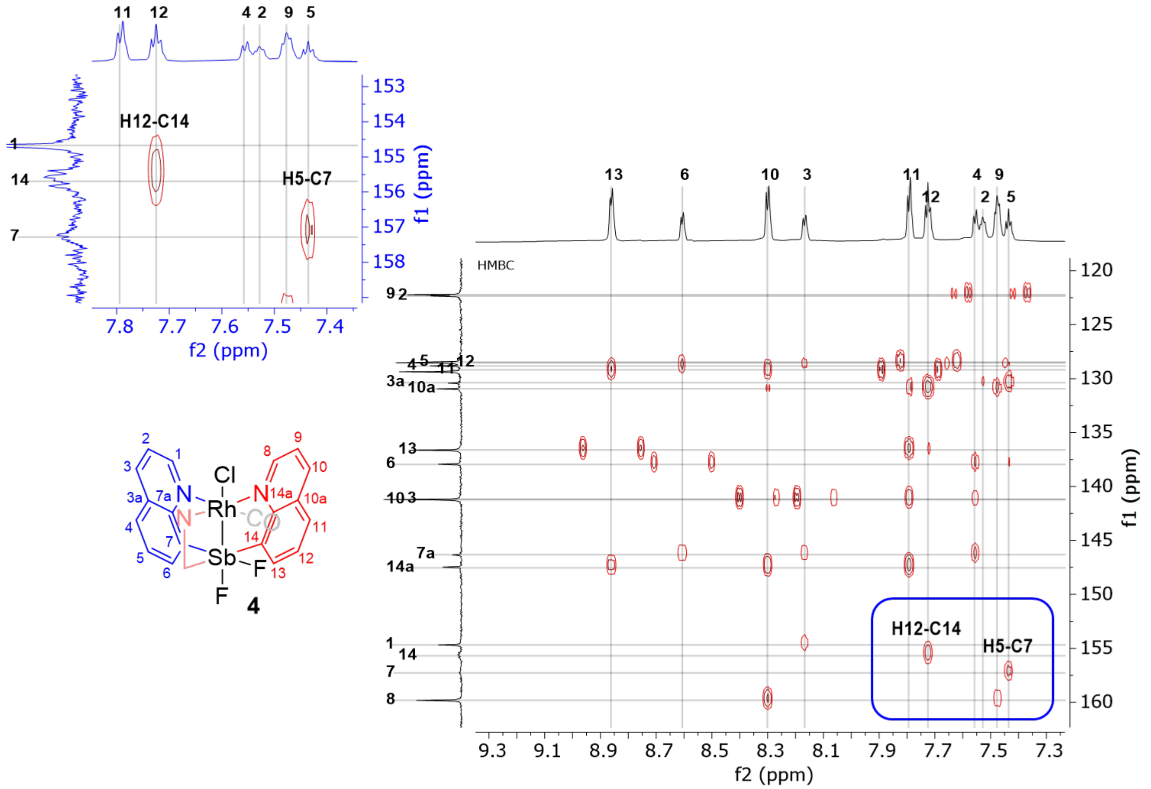

Figure S20. HMBC NMR spectrum of (κ^4-Q_3SbF_2)Rh(CO)Cl (**4**) (800, 201 MHz, CD_2Cl_2).

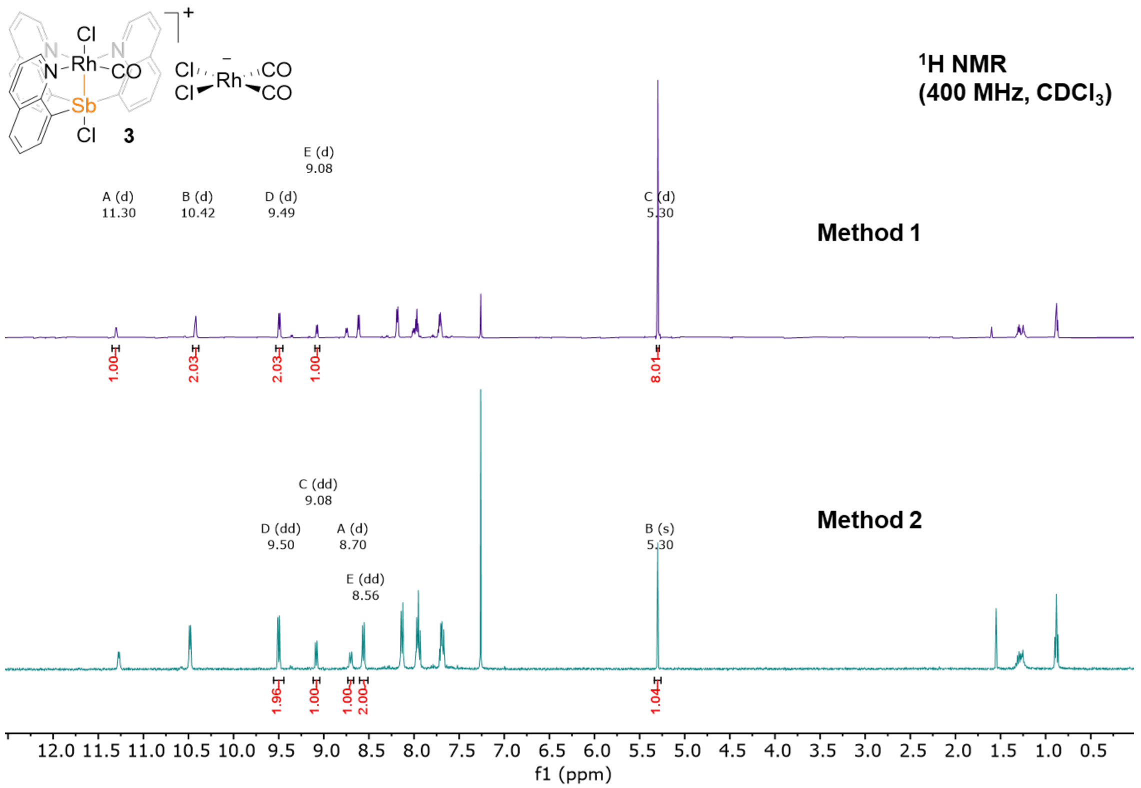

Figure S21. ^{1}H NMR spectra of recovered sample of complex **3** from elemental analysis.

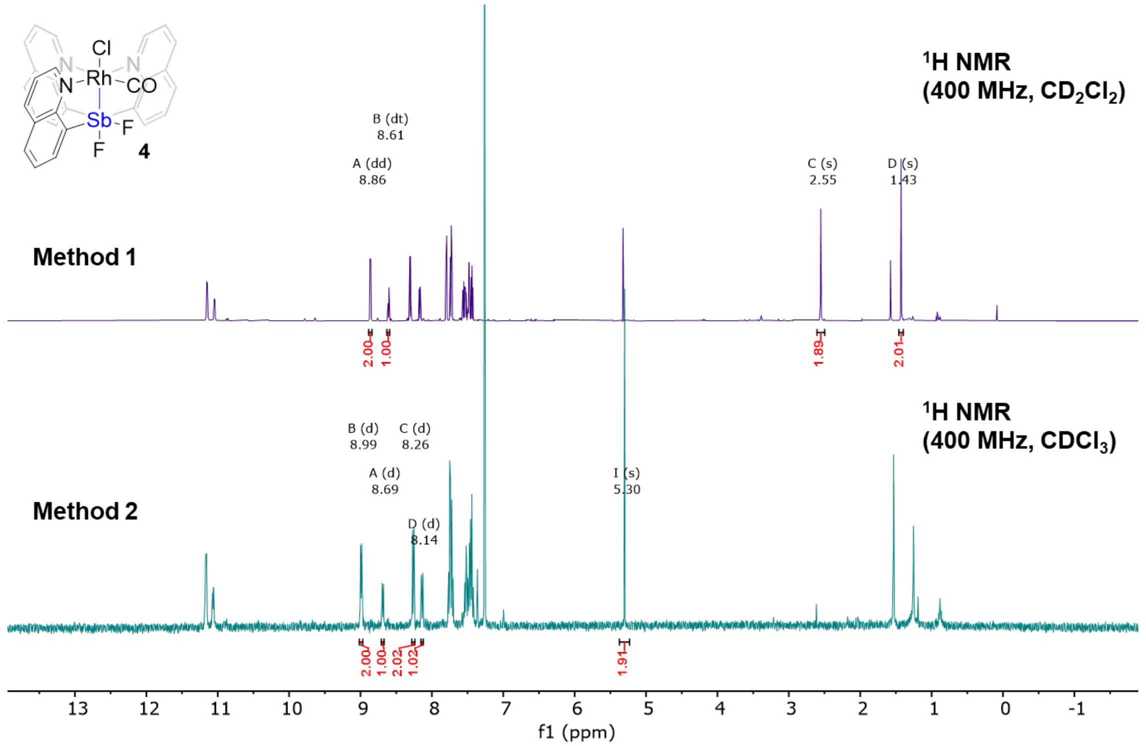

Figure S22. ^{1}H NMR spectra of recovered sample of complex **4** from elemental analysis.

6. IR Spectra

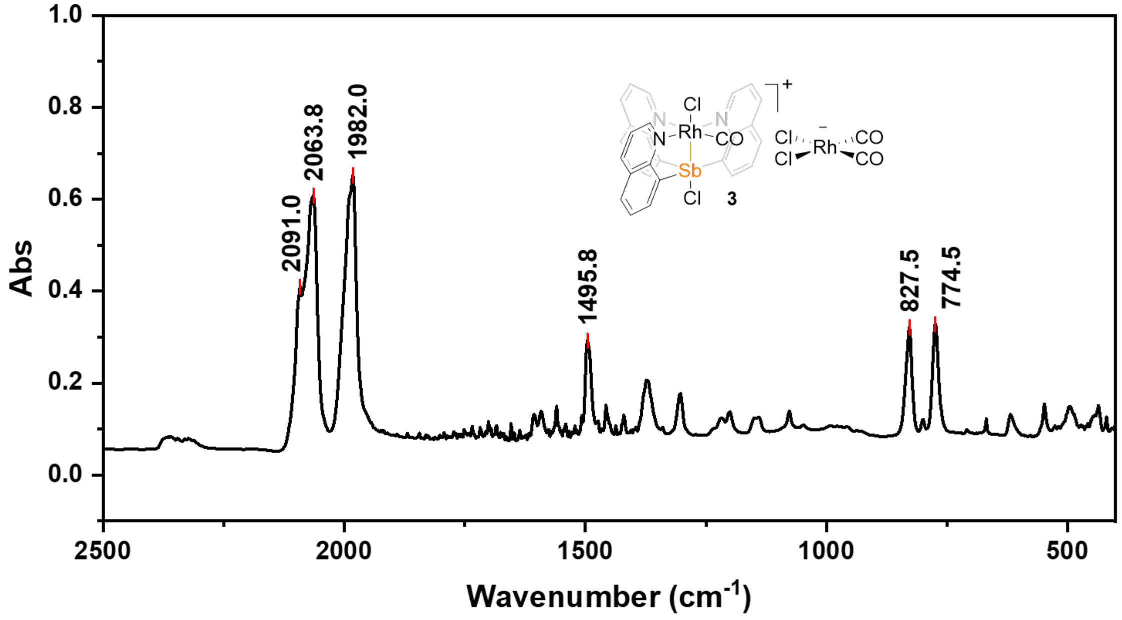

Figure S23. IR spectrum of [(κ⁴-Q_3SbCl)Rh(CO)Cl][$(CO)_2RhCl_2$] (**3**).

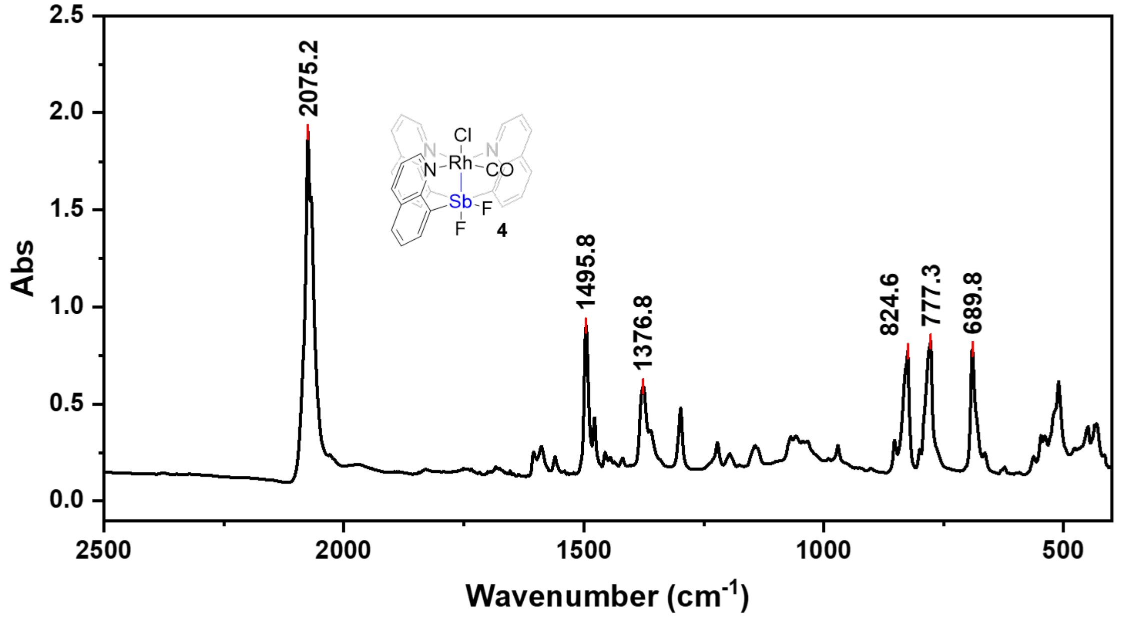

Figure S24. IR spectrum of (κ⁴-Q_3SbF_2)Rh(CO)Cl (**4**).

7. Crystal Structures of Q_3SbCl_2 and Q_3SbF_2

As shown in **Figure S25**, CH_2Cl_2 was found in the unit cell with a 1-to-1 ratio to Q_3SbCl_2 (**1**), which is consistent with the obtained elemental analysis results.

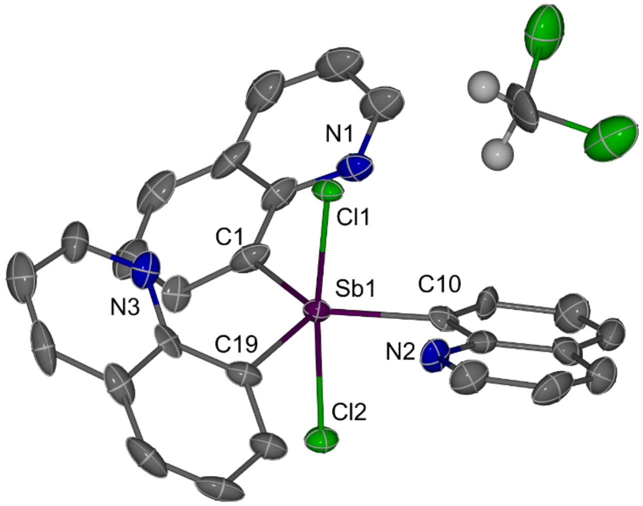

Figure S25. ORTEP of Q_3SbCl_2 (**1**). Ellipsoids are drawn at the 50% probability level and some of the hydrogen atoms are omitted for clarity.

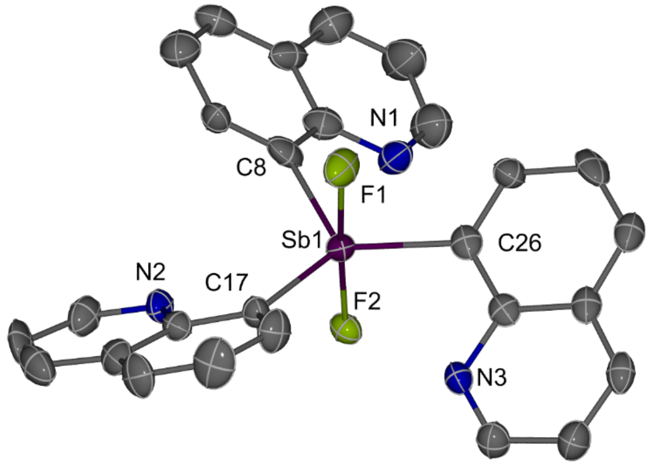

Figure S26. ORTEP of Q_3SbF_2 (**2**). Ellipsoids are drawn at the 50% probability level and hydrogen atoms and disordered chloroform solvents are omitted for clarity.

8. X-Ray Crystal Structure Data

Single crystals of all complexes were coated with Paratone oil and mounted on a MiTeGen MicroLoop. The X-ray intensity data for Q_3SbCl_2 (**1**) was measured on a Bruker Kappa APEXII Duo system equipped with a fine-focus sealed tube (Mo $K_α$, λ = 0.71073 Å) and a graphite monochromator. Data for Q_3SbF_2 (**2**), [(κ^4-Q_3SbCl)Rh(CO)Cl][$(CO)_2RhCl_2$] (**3**) and (κ^4-Q_3SbF_2)Rh(CO)Cl (**4**) were measured on a Bruker D8 VENTURE dual wavelength Mo/Cu Kappa four-circle diffractometer equipped with a PHOTON III detector. The Mo $K_α$ radiation (λ = 0.71073 Å) from an Incoatec I\mS 3.0 microfocus sealed tube with a HELIOS double bounce multilayer mirror as monochromator was used for **2** and **4**, while the Incoatec IμS 3.0 microfocus sealed X-ray tube (Cu $K_α$, λ=1.54178 Å) using a HELIOS MX double bounce multilayer mirror was used for **3**.

Data collection and processing were done within the Bruker APEX3 (**1**), APEX4 (**3**), or APEX6 (**2, 4**) software suite.[6] All data were integrated with the Bruker SAINT software using a narrow-frame algorithm. Data were corrected for absorption effects using a Multi-Scan method (SADABS or TWINABS).[6-7] Each structure was solved by dual methods with XT[8] and refined by full-matrix least-squares methods against F^2 using XL[9] within OLEX2.[10] All non-hydrogen atoms were refined anisotropically. Hydrogen atoms were placed in geometrically calculated positions with U_{iso} = 1.2U_{equiv} of the parent atom (1.5U_{equiv} for methyl). All the CIF files were generated using FinalCif.[11]

Compounds **2** and **4** were refined as twins using HKLF 5 data. For **2**, the BASF parameters of the three-domain twin refined to 0.33378 and 0.07998, and for the two-domains of **4**, the BASF was 0.46055. The relative occupancy of the disordered atoms in

2 was freely refined, using constraints on the anisotropic displacement parameters of the disordered atoms.

For complex **3**, disordered solvent located in the crystal lattice could not be adequately modeled with or without restraints. Therefore, the solvent was accounted for using the Platon SQUEEZE method.[12] A void space of 217 A^3 containing 66 electrons was found which corresponds to pentane. In the $[(CO)_2RhCl_2]^-$ anion of **3**, Cl4, C29, and O2 were disordered over two positions. The relative occupancy of the disordered atoms in the anion was freely refined with SOF of 0.768(16) / 0.232(16). Constraints and restraints were used as needed on the anisotropic displacement parameters and/or bond lengths of the disordered atoms.

Table S1. Crystal structure data table for Q_3SbCl_2 (**1**) and Q_3SbF_2 (**2**).

	**1**	**2**
CCDC number	2450879	2450880
Empirical formula	$C_{27}H_{18}N_3SbCl_2 \cdot CH_2Cl_2$	$C_{28}H_{19}Cl_3F_2N_3Sb$
Formula weight	662.02	663.56
Temperature [K]	100(2)	100.00
Wavelength [Å]	0.71073	0.71073
Crystal size [mm^3]	0.070 × 0.240 × 0.241	0.034 × 0.069 × 0.13
Crystal habit	colorless plate	plate
Crystal system	orthorhombic	orthorhombic
Space group	Pna2$_1$	Pbca
a [Å]	23.114(5)	16.7444(8)
b [Å]	13.937(3)	17.2840(7)
c [Å]	8.2207(18)	17.5899(9)
α [°]	90	90
β [°]	90	90
γ [°]	90	90
Volume [Å^3]	2648.2(10)	5090.7(4)
Z	4	8
ρ_{calc} [g·cm^{-3}]	1.661	1.732
μ [mm^{-1}]	1.469	1.438
F(000)	1312	2624
2θ range [°]	3.42 to 53.41	4.10 to 50.79
Index ranges	−28 ≤ h ≤ 29<br>−17 ≤ k ≤ 17<br>−10 ≤ l ≤ 9	0 ≤ h ≤ 20<br>0 ≤ k ≤ 20<br>0 ≤ l ≤ 21
Reflections collected	30276	7932
Independent reflections	5149<br>[R_{int} = 0.1100]	7932<br>[R_{int} = 0.1103]
Data / Restraints / Parameters	5149 / 1 / 325	7932 / 0 / 343
Goodness-of-fit on F^2	1.031	1.041
Final R indexes [I≥2σ(I)]	R_1 = 0.0460<br>wR_2 = 0.0794	R_1 = 0.0641<br>wR_2 = 0.1354
Final R indexes [all data]	R_1 = 0.0912<br>wR_2 = 0.0925	R_1 = 0.1098<br>wR_2 = 0.1578
Largest peak/hole [eÅ^{-3}]	0.806 / −0.845	1.11 / −0.67

Table S2. Crystal structure data table for [(κ[4]-Q_3SbCl)Rh(CO)Cl][$(CO)_2RhCl_2$] (**3**) and (κ[4]-Q_3SbF_2)Rh(CO)Cl (**4**).

	**3**	**4**
CCDC number	2450877	2450878
Empirical formula	$C_{30}H_{18}Cl_4N_3O_3Rh_2Sb$	$C_{30}H_{22}Cl_3F_2N_3ORhSb$
Formula weight	937.84	809.51
Temperature [K]	100.00	100.00
Wavelength [Å]	1.54178	0.71073
Crystal size [mm^3]	0.015 × 0.015 × 0.171	0.04×0.066×0.115
Crystal habit	Yellow needle	block
Crystal system	Monoclinic	triclinic
Space group	$P2_1/c$	P-1
a [Å]	9.6230(3)	9.8917(5)
b [Å]	22.5253(6)	10.1368(6)
c [Å]	17.0242(6)	15.0951(8)
α [°]	90	92.470(2)
β [°]	96.419(2)	97.844(2)
γ [°]	90	108.315(2)
Volume [$Å^3$]	3667.0(2)	1417.51(13)
Z	4	2
ρ_{calc} [$g \cdot cm^{-3}$]	1.699	1.897
μ [mm^{-1}]	15.947	1.862
F(000)	1808	792
2θ range [°]	6.53 to 136.92	4.25 to 52.96
Index ranges	$-11 \le h \le 11$<br>$-26 \le k \le 27$<br>$-19 \le l \le 20$	$-12 \le h \le 12$<br>$-12 \le k \le 12$<br>$0 \le l \le 18$
Reflections collected	30689	5856
Independent reflections	6740<br>[R_{int} = 0.1230]	5856<br>[R_{int} = 0.1146]
Data / Restraints / Parameters	6740 / 2 / 410	5856 / 0 / 381
Goodness-of-fit on F^2	1.011	1.027
Final R indexes [$I \geq 2\sigma(I)$]	R_1 = 0.0549<br>wR_2 = 0.1319	R_1 = 0.0468<br>wR_2 = 0.0885
Final R indexes [all data]	R_1 = 0.0860<br>wR_2 = 0.1474	R_1 = 0.0792<br>wR_2 = 0.1015
Largest peak/hole [$eÅ^{-3}$]	1.63 / −1.29	1.24 / −0.86

9. Computational Details and Discussion Based on Resonance Structures

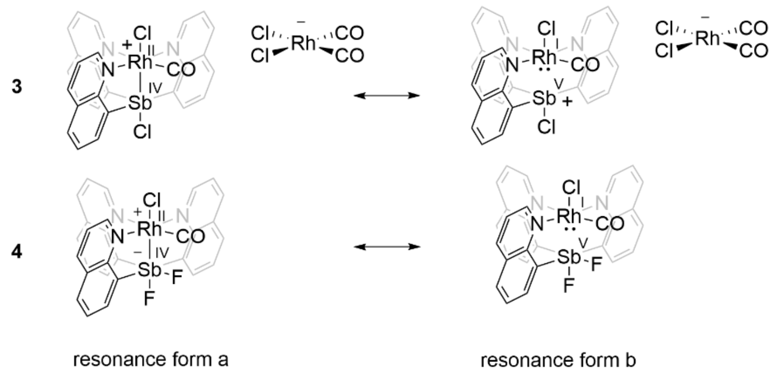

Figure S27. Resonance structures of complex **3** and **4**.

The results of calculations suggest that two main resonance forms (a and b, **Figure S27**) contribute to the bonding. Resonance form a corresponds to a complex with covalent bonding interaction between Rh–Sb. Resonance form b corresponds to a complex with electrons polarized to the Rh center. Based on calculations, complex **4** has increased contribution from resonance form b compared to **3**, which suggests that the Sb center in **4** better classified as a Z-type ligand.

Computational Methods

DFT calculations were conducted using Gaussian 16.[13] Single point energy calculations were carried out on the X-ray crystal structure geometries with the M06-L[14] meta-GGA functional and a basis set of def2-TZVP[15] basis set. All calculations were carried out in the gas phase. QTAIM[16] analysis was performed with the Multiwfn[17-18] software package and the Rh-Sb-N plane surface was plotted as the Laplacian of electron density ($\nabla^2\rho$). NBO analysis was performed with NBO 3.1[19] included with Gaussian 16.

Analysis of Multiplicity

Complexes **3** and **4** were calculated at multiple spins, including singlet triplet and quintet states; **3** was also calculated at a charge of +1 without the Rh counterion present in the X-ray structure. The lowest energy state was found to be the singlet spin state for both **3** and **4** and no internal wavefunction instabilities were found.

Table S3. Energies of **3** and **4** at different spin states. All energies are in Hartree.

**3**	-2588.788739	-2588.69425	-2588.586048
**4**	-2328.547395	-2328.43214	-2328.348961

Canonical Molecular Orbitals (MOs) and Natural Bond Orbitals (NBOs)

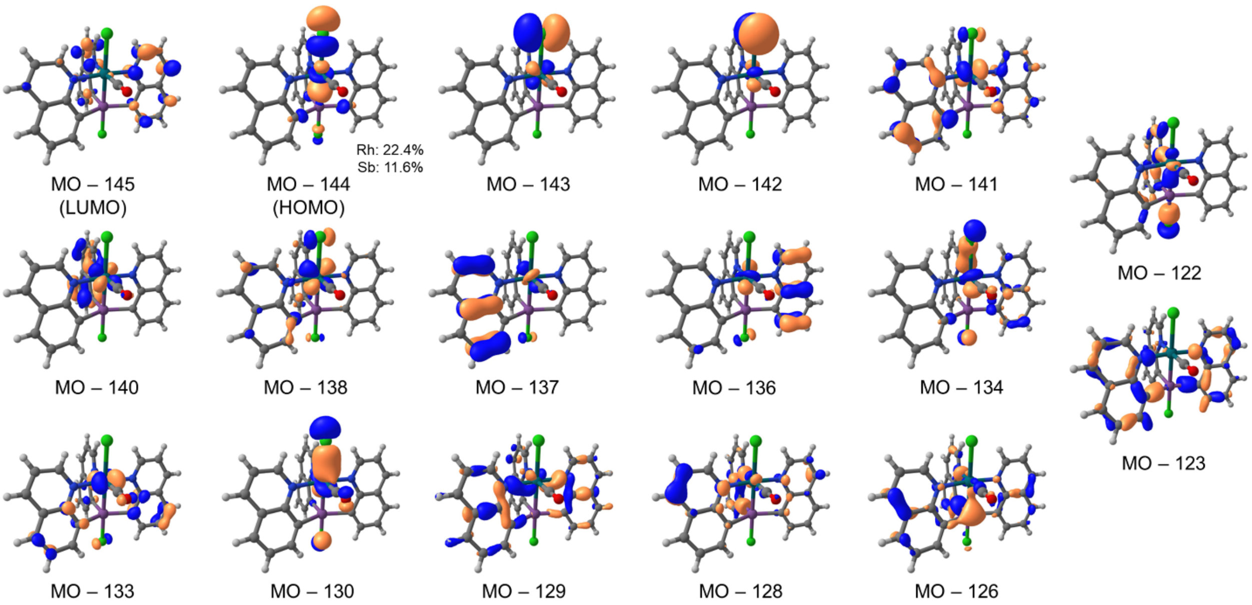

Figure S28. Canonical molecular orbitals (MOs) involving bonds with Rh and Sb for **3**.

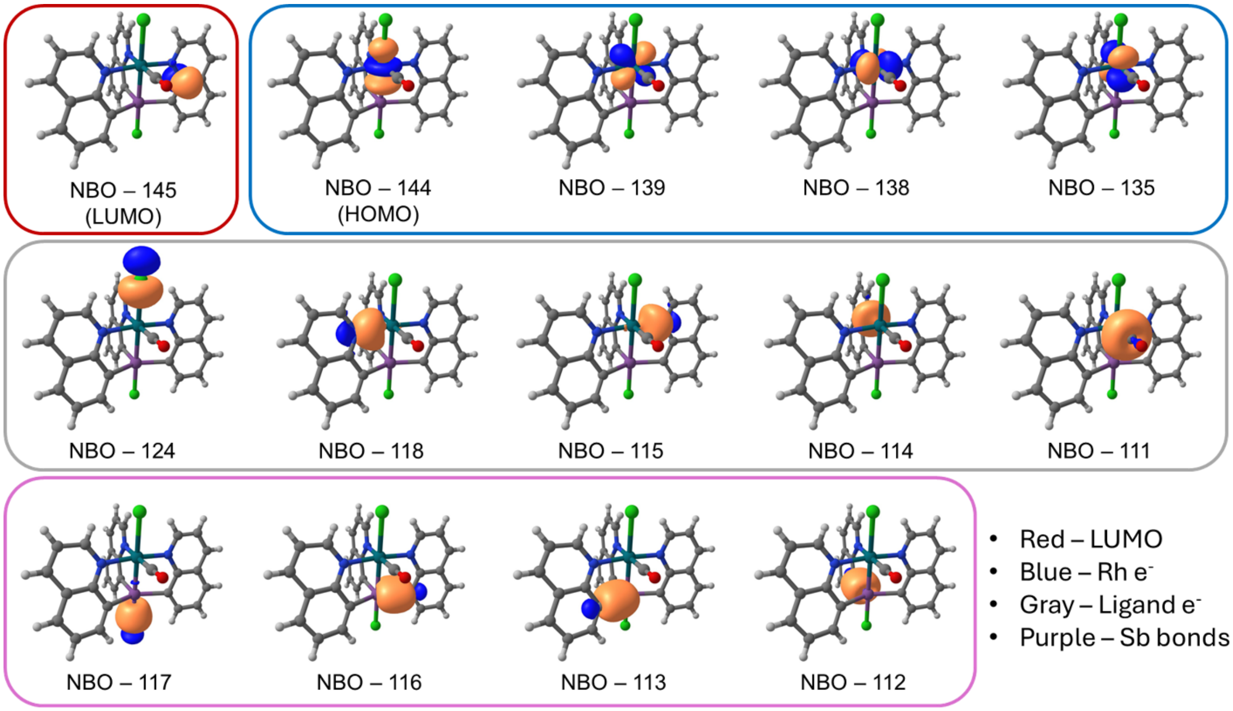

Figure S29. Natural bond orbitals (NBOs) involving bonds with Rh and Sb for **3**, highlighting different types of electrons.

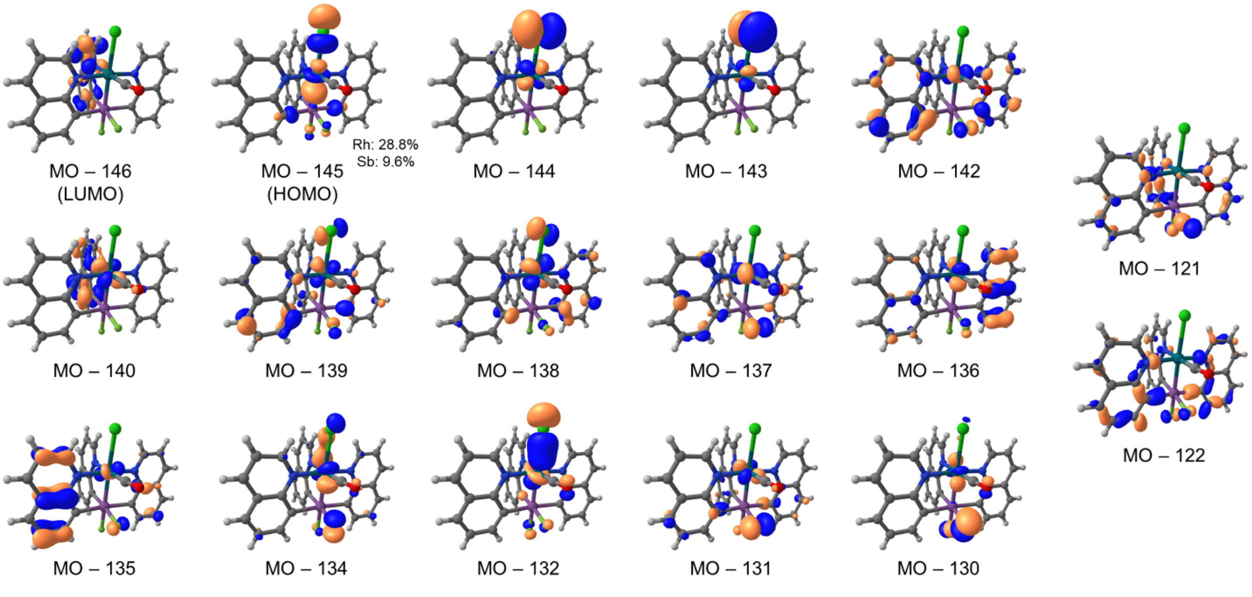

Figure 30. Canonical molecular orbitals (MOs) involving bonds with Rh and Sb for **4**.

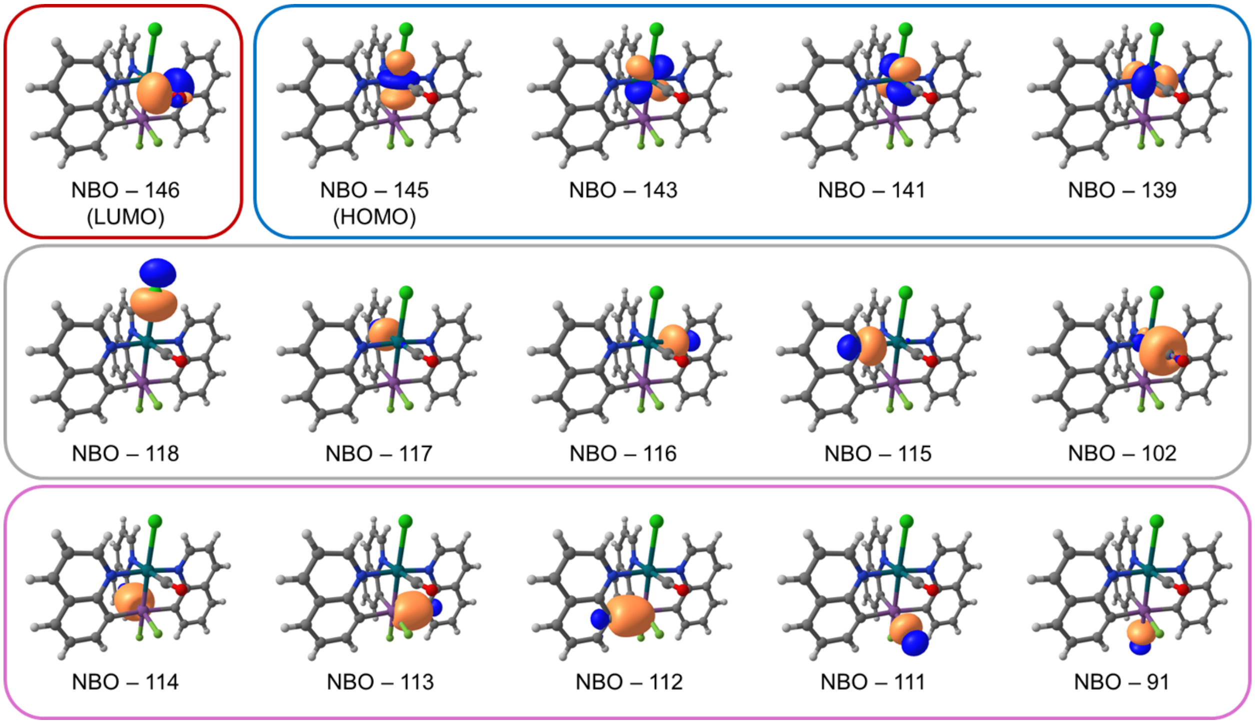

Figure S31. Natural bond orbitals (NBOs) involving bonds with Rh and Sb for **4**, highlighting different types of electrons.

10. References

1. Zhang, L.; Qi, L.; Chen, J.-M.; Dong, W.; Fang, Z.-Y.; Cao, T.-Y.; Li, W.; Wang, L.-J., Preparation of selenyl 1,3-oxazines via PhICl2/Cu2O-promoted aminoselenation of O-homoallyl benzimidates with diselenides. *Chem. Commun.* **2021,** *57*, 12655-12658. doi: 10.1039/D1CC04854F

2. Sarie, J. C.; Thiehoff, C.; Mudd, R. J.; Daniliuc, C. G.; Kehr, G.; Gilmour, R., Deconstructing the Catalytic, Vicinal Difluorination of Alkenes: HF-Free Synthesis and Structural Study of p-TolIF2. *J. Org. Chem.* **2017,** *82*, 11792-11798. doi: 10.1021/acs.joc.7b01671

3. Webber, C. K.; Kong, F.; Kumawat, J.; Joy, J.; Richardson, E. K.; Siano, P.; Dickie, D. A.; Ess, D. H.; Gunnoe, T. B., Synthesis of Quinoline-Based Pt-Sb Complexes with L- or Z-Type Interaction: Ligand-Controlled Redox via Anion Transfer. *Organometallics* **2024,** *43*, 1789-1802. doi: 10.1021/acs.organomet.4c00221

4. Fulmer, G. R.; Miller, A. J. M.; Sherden, N. H.; Gottlieb, H. E.; Nudelman, A.; Stoltz, B. M.; Bercaw, J. E.; Goldberg, K. I., NMR Chemical Shifts of Trace Impurities: Common Laboratory Solvents, Organics, and Gases in Deuterated Solvents Relevant to the Organometallic Chemist. *Organometallics* **2010,** *29*, 2176-2179. doi: 10.1021/om100106e

5. Wade, C. R.; Murphy, B. L.; Bedajna, S.; Gabbaï, F. P., Rh → Sb Interactions Supported by Tris(8-quinolyl)antimony Ligands. *Organometallics* **2024,** *43*, 1785–1788. doi: 10.1021/acs.organomet.4c00258

6. Bruker *Saint; SADABS; APEX.*, Bruker AXS Inc.: Madison, Wisconsin, USA., 2012.

7. Sevvana, M.; Ruf, M.; Usón, I.; Sheldrick, G. M.; Herbst-Irmer, R., Non-merohedral twinning: from minerals to proteins. *Acta Crystallogr. Sect. D. Biol. Crystallogr.* **2019,** *75*, 1040-1050. doi: 10.1107/s2059798319010179

8. Sheldrick, G. M., *SHELXT* – Integrated space-group and crystal-structure determination. *Acta Cryst. Sect. A Found. Adv.* **2015,** *71*, 3-8. doi: 10.1107/s2053273314026370

9. Sheldrick, G. M., Crystal structure refinement with *SHELXL*. *Acta Cryst. Sect. C Struct. Chem.* **2015,** *71*, 3-8. doi: 10.1107/s2053229614024218

10. Dolomanov, O. V.; Bourhis, L. J.; Gildea, R. J.; Howard, J. A. K.; Puschmann, H., OLEX2: a complete structure solution, refinement and analysis program. *J. Appl. Crystallogr.* **2009,** *42*, 339-341. doi: 10.1107/S0021889808042726

11. Kratzert, D. FinalCif. https://dkratzert.de/finalcif.html.

12. Spek, A. L., PLATON SQUEEZE: a tool for the calculation of the disordered solvent contribution to the calculated structure factors. *Acta Crystallogr. Sect. C: Struct. Chem.* **2015,** *71*, 9-18. doi: 10.1107/s2053229614024929

13. Frisch, M. J.; Trucks, G. W.; Schlegel, H. B.; Scuseria, G. E.; Robb, M. A.; Cheeseman, J. R.; Scalmani, G.; Barone, V.; Petersson, G. A.; Nakatsuji, H.; Li, X.; Caricato, M.; Marenich, A. V.; Bloino, J.; Janesko, B. G.; Gomperts, R.; Mennucci, B.; Hratchian, H. P.; Ortiz, J. V.; Izmaylov, A. F.; Sonnenberg, J. L.; Williams; Ding, F.; Lipparini, F.; Egidi, F.; Goings, J.; Peng, B.; Petrone, A.; Henderson, T.; Ranasinghe, D.; Zakrzewski, V. G.; Gao, J.; Rega, N.; Zheng, G.; Liang, W.; Hada, M.; Ehara, M.; Toyota, K.; Fukuda, R.; Hasegawa, J.; Ishida, M.; Nakajima, T.; Honda, Y.; Kitao, O.; Nakai, H.; Vreven, T.; Throssell, K.; Montgomery Jr., J. A.; Peralta, J. E.; Ogliaro, F.; Bearpark, M. J.; Heyd, J. J.; Brothers, E. N.; Kudin, K. N.; Staroverov, V. N.; Keith, T. A.; Kobayashi, R.; Normand, J.; Raghavachari, K.; Rendell, A. P.; Burant, J. C.; Iyengar, S. S.; Tomasi, J.; Cossi, M.; Millam, J. M.; Klene, M.; Adamo, C.; Cammi, R.; Ochterski, J. W.; Martin, R. L.; Morokuma, K.; Farkas, O.; Foresman, J. B.; Fox, D. J. *Gaussian 16 Rev. C.01*, Wallingford, CT, 2016.

14. Zhao, Y.; Truhlar, D. G., A new local density functional for main-group thermochemistry, transition metal bonding, thermochemical kinetics, and noncovalent interactions. *J. Chem. Phys.* **2006,** *125*, 194101. doi: 10.1063/1.2370993

15. Weigend, F.; Ahlrichs, R., Balanced basis sets of split valence, triple zeta valence and quadruple zeta valence quality for H to Rn: Design and assessment of accuracy. *Phys. Chem. Chem. Phys.* **2005,** *7*, 3297-3305. doi: 10.1039/B508541A

16. Bader, R. F. W., A quantum theory of molecular structure and its applications. *Chem. Rev.* **1991,** *91*, 893-928. doi: 10.1021/cr00005a013

17. Lu, T.; Chen, F., Multiwfn: A multifunctional wavefunction analyzer. *J. Comput. Chem.* **2012,** *33*, 580-592. doi: 10.1002/jcc.22885

18. Lu, T., A comprehensive electron wavefunction analysis toolbox for chemists, Multiwfn. *J. Chem. Phys.* **2024,** *161*. doi: 10.1063/5.0216272

19. Glendening, E. R., A.; Carpenter, J.; Weinhold, F. *NBO version 3.1*, Gaussian. Inc.: Pittsburgh, PA, 2003.
